# WDR-5 exhibits H3K4 methylation-independent activity during embryonic development in *C. elegans*

**DOI:** 10.1186/s13072-026-00669-y

**Published:** 2026-03-25

**Authors:** Nurulhafizah Binti Samsudin, Siyao Wang, Kate Fisher, Gino B. Poulin

**Affiliations:** 1 Division of Molecular and Cellular Function, School of Biological Sciences, Faculty of Biology, Medicine and Health, Manchester Academic Health Science Centre, University of Manchester, Oxford Road, Manchester, M13 9PL UK; 2 Institute of Molecular Biology (IMB), Ackermannweg 4, 55128 Mainz, Germany

**Keywords:** Chromatin, H3K4 methylation, MLL/SET/COMPASS complex, Chromosome X, WDR-5, RBBP-5, *C. elegans* embryo

## Abstract

**Background:**

Histone H3 lysine 4 methylation (H3K4me) is generally associated with active transcription and bivalent chromatin but can also contribute to repression. In metazoans, H3K4 methylation is catalysed by KMT2 methyltransferases assembled with the core scaffolding proteins WDR5, ASH2L, and RBBP5. RBBP5 mediates complex assembly and nucleosome binding, whilst WDR5 stabilises interactions to promote tri-methylation. However, WDR5 also exhibits additional moonlighting functions, leaving its specific roles in H3K4 methylation and transcription regulation unclear. Using *C. elegans* embryos, spike-in ChIP-seq, and null alleles of *wdr-5(-)* and *rbbp-5(-)*, we dissected the contributions of these scaffolds towards H3K4 mono-, di-, and tri-methylation as well as gene expression during *C. elegans* embryogenesis.

**Results:**

We show that *C. elegans* RBBP-5 is essential for both mono- and multi-methylated H3K4 deposition. On the other hand, WDR-5 is primarily required for H3K4me3, but can influence H3K4me2 and H3K4me1 deposition either positively or negatively depending on the genomic feature involved. We additionally performed RNA-seq on these mutants and found that *rbbp-5* deletion was largely tolerated with mis-regulation of ~ 700 genes, whereas the *wdr-5* deletion led to widespread transcriptomic disruption (~ 3000 genes). We initially hypothesised that these broad changes were driven by the altered H3K4me1/me2 landscape in the *wdr-5(-)* mutant. However, transcriptomic profiling of the *wdr-5(-); rbbp-5(-)* double mutant, which lacks H3K4 methylation, revealed a high degree of similarity to the *wdr-5(-)* single mutant. This refuted our initial hypothesis and indicated that the changes in H3K4 methylation are unlikely to underlie the transcriptional effects of the *wdr-5* deletion.

**Conclusions:**

Our findings support the notion that WDR-5 profoundly shapes gene expression through RBBP-5-independent mechanisms and possibly beyond H3K4 methylation. Distinguishing between H3K4me-dependent and independent functions of WDR-5 will further understanding of its roles in development and disease.

**Supplementary Information:**

The online version contains supplementary material available at 10.1186/s13072-026-00669-y.

## Background

The chromatin landscape is shaped by the organisation of nucleosomes within the confined space of the nucleus. The resulting epigenome facilitates the organisation of RNA polymerase II into transcription factories and regulatory hubs that help establish and maintain gene expression patterns during development [1]. Histones, together with DNA, form nucleosomes, the fundamental units of chromatin. Initially described as barriers to transcription, decades of chromatin research have since revealed that nucleosomes play far more nuanced roles, acting through post-translational modifications that can either promote or repress transcription [2, 3].

One of the best-characterised histone modifications occurs on histone 3 at lysine 4 (H3K4), which can be mono-, di-, or tri-methylated (H3K4me1, H3K4me2, and H3K4me3). These three methylation states display distinct genomic distributions and are broadly associated with transcriptionally active genes [4]. Beyond transcription, H3K4 methylation also participates in replication, pre-mRNA splicing, and DNA repair [5–10]. H3K4me3 is highly enriched at active transcription start sites (TSS) [11–13] and serves as a recognition signal for specific reader proteins that can interact with the basal transcriptional machinery [14–17]. It contributes to both transcriptional initiation and RNA polymerase II pause-release during early elongation [4, 18–20]. Broader H3K4me3 domains have been linked to transcriptional consistency and the expression of tumour suppressor genes [21, 22]. At CpG islands, H3K4me3 recruits the CXXC1/CFP1 subunit of the SET1 complex, which in turn helps protect CpGs from DNA methylation [23]. Although *C. elegans* lacks canonical CpG methylation, CFP-1 is conserved, suggesting it mediates analogous chromatin-activating functions [24]. H3K4me2 and H3K4me3 can also participate in the formation of bivalent domains when co-occurring with H3K27me3. These domains mark developmental genes that are repressed yet poised for later activation [25]. In addition, H3K4me2 can mediate non-coding RNA-dependent repression of Hox genes [26], while both H3K4me2 and H3K4me3 help suppress cryptic transcription within gene bodies [27–30]. Finally, enrichment of H3K4me1 together with H3K27ac defines transcriptionally active enhancers [31, 32]. Collectively, these studies indicate that alterations in H3K4 methylation can compromise transcriptional homeostasis by disrupting both activating and repressive chromatin mechanisms.

H3K4 methylation is catalysed by the evolutionarily conserved SET/COMPASS and MLL (Mixed Lineage Leukaemia) complexes [33–35] referred to hereafter collectively as the SET/MLL complex. These complexes consist of SET domain-containing methyltransferases (KMT2 enzymes) and a core scaffolding complex that together transfer methyl groups from the universal donor S-adenosylmethionine to H3K4, generating H3K4me1/me2/me3 [36]. Notably, the number of KMT2 enzymes increases with species complexity: one in *S. cerevisiae*; two in *C. elegans*; three in *Drosophila*; and six in mammals [37, 38]. These enzymes form distinct SET/MLL sub-complexes that share core components but also include specific co-factors. The main core subunits are WDR5, RBBP5, ASH2L, and DPY30, which associate with co-factors such as CFP1 or Menin to form SET or MLL complexes, respectively [39]. Interestingly, WDR5 is also found in other chromatin-modifying complexes containing HAT or HDAC activities [24, 40–46], challenging the attribution of WDR5-dependent transcriptional changes solely to its role in H3K4 methylation.

Structural and biochemical studies in yeast and humans have demonstrated that the scaffolding subunits WDR5 and RBBP5 are not functionally equivalent. RBBP5 acts as a central linchpin of the SET/MLL complex through its WD40 propeller, which directly contacts the nucleosome and cooperates with ASH2L to stimulate the catalytic activity of KMT2 enzymes [6, 36, 47, 48]. In contrast, WDR5 plays more nuanced roles: it promotes the assembly of MLL1-containing complexes in humans, is dispensable for activation of several other MLL family members, and can exert opposing effects depending on the specific MLL paralogue involved [36, 47]. These structural distinctions suggest that the transcriptional defects and alterations in H3K4 methylation arising from loss of WDR-5 or RBBP-5 should be distinct, yet this has not been systematically tested in metazoans.

In *C. elegans*, the SET/MLL complex comprises two KMT2 enzymes: SET-2, a SET1 orthologue [49] and SET-16, an MLL-like enzyme [50]. The scaffolding subunits WDR-5, ASH-2, and RBBP-5 are highly conserved across species [49–52]. Independent loss of these scaffolding components leads to phenotypes including altered lifespan [8, 53–55], erroneous hindgut-to-neuron transdifferentiation [56], increased RAS signalling in vulval cells [50], axon guidance defects [57] and disrupted germ cell pluripotency [58, 59]. SET-16-dependent H3K4me3 also contributes to innate immunity [60].

The contributions of WDR-5 and RBBP-5 to H3K4 methylation in *C. elegans* have been examined primarily through western blotting and immunofluorescence [49, 51, 52, 61]. These studies show that RBBP-5 is required for both H3K4me2 and H3K4me3 deposition, whereas its role in H3K4me1 deposition remains to be determined. In contrast, WDR-5 is essential for H3K4me3 but dispensable for H3K4me1, while its contribution to H3K4me2 remains debated. Two studies reported approximately a 50% reduction in H3K4me2 levels [49, 61], whereas another observed a near-complete loss [51], but developmental stage (embryonic versus larval) or fixation protocols, may underlie this apparent discrepancy. Despite clear evidence that both subunits influence H3K4 methylation to varying degrees, a systematic and quantitative comparison of their individual contributions across genomic features such as transcription start sites and enhancers has yet to be performed.

**Fig. 1:**
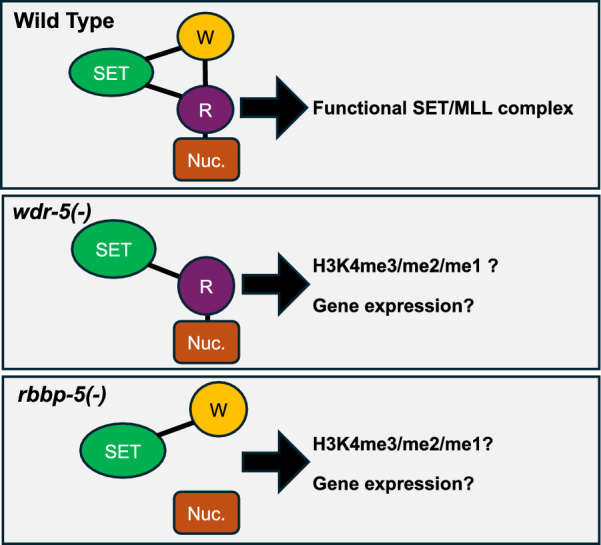
Working model of the SET/MLL complex in wild type, *wdr-5(-)*, and *rbbp-5(-)* embryos and experimental rationale. **A** Wild-type SET/MLL complex. Schematic representation of a fully assembled SET/MLL complex showing the catalytic SET domain (green), the scaffolding subunit WDR-5 (orange), and RBBP-5 (purple), which contacts the nucleosome (brown) to anchor and orient the complex. In this configuration, all three H3K4 methylation states (H3K4me1/2/3) can be efficiently deposited. **B** Loss of WDR-5. In the absence of WDR-5, the catalytic enzyme can still associate with RBBP-5 and dock onto the nucleosome. Some H3K4 methylation activity is therefore predicted to remain. **C** Loss of RBBP-5. Without RBBP-5, the SET domain is unable to stably engage the nucleosome, leading to a substantial predicted loss of H3K4 methylation activity across all methylation states. These conceptual models illustrate the basis for our experimental approach: analysing how removal of either scaffolding subunit affects H3K4 mono- and multi-methylation and the corresponding transcriptional outputs

Herein, we systematically defined the contributions of WDR-5 and RBBP-5 to all three H3K4 methylation states using spike-in-normalised ChIP-seq in *C. elegans* embryos and mapped these effects across genomic features, including transcription start sites (TSS) and enhancers (Fig. 1). We found that WDR-5 is specifically required for H3K4me3 deposition, whereas RBBP-5 is essential for the maintenance of all three H3K4 methylation states. RNA-seq analysis revealed that *rbbp-5(-)* mutant embryos exhibited markedly fewer transcriptomic changes than *wdr-5(-)* embryos (721 versus 3377 differentially expressed genes, respectively). Moreover, the transcriptome of the *rbbp-5(-); wdr-5(-)* double mutant, which lacks detectable H3K4 methylation, closely resembled that of the *wdr-5(-)* single mutant. These findings suggest that the extensive transcriptional changes observed in the absence of WDR-5 can occur independently of RBBP-5 and by extension, of H3K4 methylation. Together, our results indicate that while WDR-5 is required for H3K4me3 deposition, it also likely exerts additional, H3K4 methylation-independent functions important to regulate gene expression in embryos.

## Materials and methods

### Strains and general maintenance

*C. elegans* strains were maintained at 20 °C and as described in Brenner et al. [62]. Strains used in this study are: N2 (wild type), *wdr-5.1(ok1417) III* (RB1304), *rbbp-5(tm3463) II,* and the double *rbbp-5(tm3463) II; wdr-5.1(ok1417) III* (OL76).

### Embryonic preparation on large solid media plates for spike-in ChIP-seq

Synchronised population of embryos were prepared on solid NGM media from bleached young adult hermaphrodites. These adults were harvested from two rounds of amplifications; each performed on 20 Corning tissue culture dishes (245 × 245 mm) that were seeded with synchronised L1 (larval stage 1) obtained from bleaching large numbers of gravid adults. To obtain these large populations of worms, dishes were seeded with 0.5 ml of 20 × OP50 stock and left to dry for at least two days before use. 20 dishes per strain were seeded with synchronised L1s: ~ 1,000 L1s for N2 (wild type), ~ 2,000 L1s for *wdr-5(-)*, and ~ 3,000 L1s for *rbbp-5(-)*. When the next generation reached L4 stage, all worms were harvested and transferred onto 20 new dishes to prevent starvation and mature adults were bleached to generate large populations of synchronised L1s, which were all re-seeded onto 20 dishes. These L1s were grown to obtain young adults, a stage reached after 54 h for N2 (wild type), and 64 h for *wdr-5(-)* and *rbbp-5(-)*. Following bleaching of these adults, embryos were collected on sucrose gradients and re-suspended in 47 ml of M9 buffer and 2.8 ml of 37% formaldehyde solution (~ 2% final). Embryos were placed on a shaker (50 rpm) at room temperature for 30 min, then spun down and the pellets quenched by adding 50 ml of 100 mM Tris buffer (pH 7.5) to stop the fixation process. Fixed embryos were spun down and washed twice with 50 ml M9, then 10 ml FA buffer (50 mM HEPES/KOH pH 7.5, 1 mM EDTA pH 8, 1% Triton x-100, 0.1% sodium deoxycholate, 150 mM NaCl) containing protease inhibitor cocktail was added to the pellet. Between 200 l and 500 l of packed embryos can be obtained from this method. Next, an aliquot of 5 l of embryos for each genotype was taken out, treated with methanol, and stained with DAPI for scoring on standard 2% agarose pad with a coverslip (store at − 20 °C). The embryo preparations used for N2 (wild type), *wdr-5(-)*, and *rbbp-5(-)* were comparable: 20–40% at < 28-cell stages; 20–27% between 28-cell and 100-cell stages; 32–52% at pre-coma stage; and 0–4% at coma stage or beyond. Then, samples were prepared as previously described [63]. Briefly, embryo pellets were re-suspended in a total of 1.5 ml FA buffer (50 mM HEPES/KOH pH 7.5, 1 mM EDTA pH 8, 1% Triton x-100, 0.1% sodium deoxycholate, 150 mM NaCl) with protease inhibitor cocktail. Embryos were dounced 40 times on ice using a pastel B homogeniser and aliquoted in 250 l into six 1.5 ml microtubes. Sonication was performed using the Bioruptor UCD-200 (30 s ON, 30 s OFF) with ultrasonic wave output at HIGH for three 5 min cycles. Between cycles, the samples were cooled in a dry ice/ethanol bath for 5 s. A small aliquot was kept to verify the DNA size on a 1.5% agarose gel showing enrichment between 400–600 bp. In the meantime, the samples were snap frozen and kept at -80 °C. The frozen embryonic samples were then shipped to ActiveMotif on dry ice to perform the spike-in ChIP-seq procedure [64]. Briefly, about 5% of *D. melanogaster* was added to *C. elegans* embryo chromatin. 0.4 μg of the anti-H2Av antibody (AM39715) was used to immunoprecipitate *D. melanogaster* chromatin from each reaction, which is then used as a reference for normalisation. The antibodies are from ActiveMotif and are: anti-H3K4me1 (AM39297), anti-H3K4me2 (AM39141), and anti-H3K4me3 (AM39159).

### Spike-in ChIP-seq libraries and data processing

Illumina sequencing libraries were prepared from the ChIP and input DNAs by the standard consecutive enzymatic steps of end-polishing, dA-addition, and adaptor ligation. After a final PCR amplification step, the resulting DNA libraries were quantified and sequenced on Illumina’s NextSeq 500 (75 nt reads, single end).

All genomic analyses were performed using the *Caenorhabditis elegans* genome build WS220 (ce10). ChIP-seq reads were aligned to the ce10 genome using Bowtie2 v2.3.4 and processed with Samtools v1.9 and Picard. Duplicate reads were removed using MarkDuplicates, and mitochondrial reads were filtered out. To account for GC content bias, we applied computeGCBias and correctGCBias from DeepTools (v3.5.6) [65]. Normalisation by downsampling was carried out on the GC-corrected BAM files using Picard and spike-in normalisation factors applied. Final normalised coverage tracks (BigWigs) were generated from the downsampled, GC-corrected BAM files using bamCoverage with CPM normalisation and a bin size of 10 bp. These tracks were used in all downstream DeepTools analyses, including heatmaps, metagene plots, and principal component analysis.

The N2 wild type data were benchmarked against modEncode publicly available early embryonic ChIP-seq dataset (H3K4me1 repl 1: GSM1217259 repl 2: GSM1217260 input: GSM1217261; H3K4me2 repl 1: GSM1206344 repl 2: GSM1206345 input: GSM1206346; H3K4me3 repl 1: GSM1206368 repl 2: GSM1206369 input: GSM1206370) [66].

To compare H3K4me1, H3K4me2, and H3K4me3 levels between wild type and mutants, Transcription Start Sites (TSS) generated by Chen et al. [67] and active enhancers mapped by Janes et al. [68] were used. Normalised bigWig files containing ChIP-seq signal coverage were analysed using Deeptools [65]. The bigWig files corresponding to wild-type (WT), *wdr-5(-)*, and *rbbp-5(-)* conditions were GC bias-corrected and normalised. BED files defining TSS and enhancer regions were uploaded for further processing.

### Peak annotation and genome-wide distribution analysis

Broad peak regions were identified using MACS2 (v2.2.9.1) in –broad mode (–broad-cutoff 0.025, -q 0.005) on GC-corrected, downsampled ChIP-seq BAM files with matched input controls (effective genome size 9.3 × 10⁷ bp). Peaks were post-filtered using mark-specific thresholds: H3K4me1/2 peaks were retained if length ≥ 2 kb, signalValue ≥ 3, and q ≤ 0.005; H3K4me3 peaks were retained if length ≥ 0.5 kb, signalValue ≥ 3, and q ≤ 0.005. Filtered peaks were converted to BED format and intersected with genomic annotations using ChIPseeker and GenomicRanges in R. A custom TxDb object was generated from the *C. elegans* ce10 reference genome (txdbmaker::makeTxDbFromGFF), and additional features were incorporated to increase resolution, including transcription start sites (TSS) and enhancer coordinates. Each peak was classified into four mutually exclusive categories: Promoter_only (overlaps a TSS region ± 1 kb but not an enhancer), Enhancer_only (overlaps an enhancer but not a TSS region), Promoter_Enhancer (overlaps both TSS and enhancer coordinates), and Other (peaks outside annotated regulatory regions). Counts, cumulative signal intensities (signalValue from MACS2), and cumulative area (signal × width) were computed per category for each genotype (N2, *wdr-5(-)*, *rbbp-5(-)*) and each methylation state (H3K4me1/2/3).

### Chromosome-level bias assessment of input samples

To verify that Input chromatin coverage was uniform across chromosomes, we quantified genome-wide read distribution using RPGC-normalised bigWig tracks generated with deepTools bamCoverage (v3.5.1). Chromosome sizes (ce10) were extracted from the BAM index, and a BED file containing one interval per chromosome (chrI–chrX) was used with multiBigwigSummary to compute mean RPGC signal per chromosome. Log₂(observed/expected) coverage was then calculated, and global uniformity was assessed using a chromosome-size-weighted chi^2^ goodness-of-fit test. X-chromosome behaviour was evaluated using the log_2_ ratio of chrX signal relative to autosomes.

### Western blots

Western blot analysis was performed as described in [50] with a few modifications. Briefly, embryo protein extracts were prepared by bleaching young mothers followed by washing the embryos four times with M9 buffer. A minimum volume of 20 μl pelleted embryos was collected for each protein sample, boiled in Laemmli buffer containing 100 mM DTT and sonicated. Western blot films were scanned and analysed using Fiji. For each lane, a region of interest of identical size was drawn using the rectangle tool for each band, and local background was measured from an adjacent region of identical size below the band and subtracted from the band intensity. Histone H3 was quantified using the same approach and used for normalisation. Mutant samples were quantified relative to wild-type control on the same membrane. Quantification was performed on three independent biological replicates.

### Immuno-fluorescence assay

Immuno-fluorescence was performed by freeze crack method as described in [52]. Briefly, mothers were placed in M9 buffer and embryos were released by dissection, frozen, methanol fixed, washed in PBS-tween 0.2% and exposed to antibodies and DAPI for staining. Images were quantified using ImageJ software.

### Embryonic preparations for RNA-seq

Synchronised populations of adult worms for N2, *rbbp-5(-)*, *wdr-5(-)* and *rbbp-5(-); wdr-5(-)* were grown on 10 cm plates. Young adults were bleached and the embryos scored for developmental stages using DIC microscopy. The populations were consistently between ~ 25–30% at 28–100 cell stage, ~ 45–55% at pre-coma stage, and ~ 5–15% at late embryogenesis stages, roughly matching the spike-in ChIP-seq samples. Three biological replicates were prepared per genotype. Total RNA was extracted using TRIzol (Invitrogen) and stored at -80 °C until sent for analysis to the Genomic core facility at the Faculty of Biology, Medicine and Health (University of Manchester).

### RNA-seq libraries and analysis

RNA-seq libraries were generated using the TruSeq Stranded mRNA Sample Preparation Kit (Illumina), followed by 101 × 101 bp paired-end sequencing on the Illumina HiSeq platform. Across 12 libraries, an average of ~ 208 million paired-end reads per sample were obtained (range: 100–422 million), with an average alignment rate of 94% to the *C. elegans* reference genome (ce10). Mate 1 and mate 2 reads were balanced across all samples, indicating high-quality libraries. FastQC and Trimmomatic [69] were used for quality control and adapter trimming. Reads were aligned using TopHat 2.1.0 [70], and gene-level quantification was performed using HTSeq [71] with the c_elegans WS220.annotations.gtf annotation file. Differentially expressed genes (DEG) were identified using DESeq2 [72], applying thresholds of fold change > 2 and adjusted p-value < 0.05 with a cut-off for read counts set at 50 as detected in wild type and/or mutant samples.

### Quantitative RT-PCR

Embryo pellets were prepared by harvesting synchronised gravid adults, washing the pellet in M9 and bleaching as routine. Total RNA was extracted using TRIzol (Invitrogen) and samples were kept at -80 °C. First strand cDNA synthesis was performed using the SuperScript VILO cDNA Synthesis Kit (Invitrogen), according to the manufacturer's instructions. Quantitative RT-PCR was performed using the FastStart SYBR Green Master (ROX) mix (Roche) on a Biorad C1000 thermal cycler coupled with a CFX96 Real time system. Three biological samples from each strain were prepared and analysed in triplicate by the ΔΔCT method [73]. *act-1* was used as the internal reference for data normalisation (see Table S8 for primers).

### Calculation of net transcriptome change percent

To assess the global directional impact of transcriptomic changes in mutants relative to wild type, we calculated the Net Transcriptome Change Percent by integrating the direction of expression change (log₂ fold change) with the level of gene expression (baseMean) for differentially expressed genes (DEG). Only DEG passing thresholds for both adjusted p-value and fold change were included in this analysis. For each misregulated gene, the baseMean (mean normalised expression across all samples) was multiplied by its log₂-transformed fold change (log₂FC). The contributions of all misregulated genes were summed to produce the Net Directional Change. The baseMean values for all DEG were summed to calculate the Total BaseMean, representing the total transcript abundance for all misregulated genes irrespective of regulation direction. The Net Transcriptome Change Percent was calculated by expressing the Net Directional Change as a proportion of the Total BaseMean and multiplied by 100. This metric provides a global estimate of the directional bias in transcriptomic change, expressed as a percentage of the total misregulated gene expression. Negative percentages indicate net down-regulation; positive percentages indicate net up-regulation.

### Statistical test on the violin plots

We tested whether genes classified as upregulated, down-, or misregulated in a mutant background tend to have different expression levels in wild type compared to unregulated genes, using the baseMeanA metric (representing average wild-type expression). We performed a Mann–Whitney U test, also called a Pairwise Wilcoxon rank-sum tests, applied to log2-transformed baseMeanA values to assess distributional shifts across categories.

### Cook’s distance analysis

To assess replicate variability and potential batch effect in the *rbbp-5(-)* RNA-seq dataset, differential expression analysis was performed using DESeq2 with an explicit batch term. Taw gene-level count matrices were analysed using a generalised linear model incorporating both batch and genotype. Cook’s distance was extracted from the fitted DESeq2 model to identify genes for which individual samples exerted a disproportionate influence on model estimates. Genes with Cook’s distance > 1 in the rbbp5_1 replicate were identified and intersected with the full *rbbp-5(-)* differential expression results table. To ensure consistency with the primary analysis, the same expression filter was applied, retaining only genes with baseMean values > 50 in both wild type and *rbbp-5(-)* conditions. Differential expression significance was assessed using the original thresholds (|log2 fold change|> 1 and adjusted p-val < 0.05). As a diagnostic test, relaxed thresholds (|log2 fold change|> 0.75 and adjusted p-val < 0.1) were also applied. To visualise the distribution of effect sizes and statistical significance among Cook’s-sensitive gens, a two-dimensional point-density plot was generated showing log2 fold change versus p-adj, with colour coded dots reflecting local gene density estimated by kernel density smoothing.

### ChIP-seq and RNA-seq data access

The ChIP-seq data have been deposited in the GEO repository under ID code GSE94639 and RNA-seq data have been deposited in the ArrayExpress repository under ID code E-MTAB-15080.

## Results

### Benchmarking H3K4 mono- and multi-methylation spike-in ChIP-seq data

**Fig. 2:**
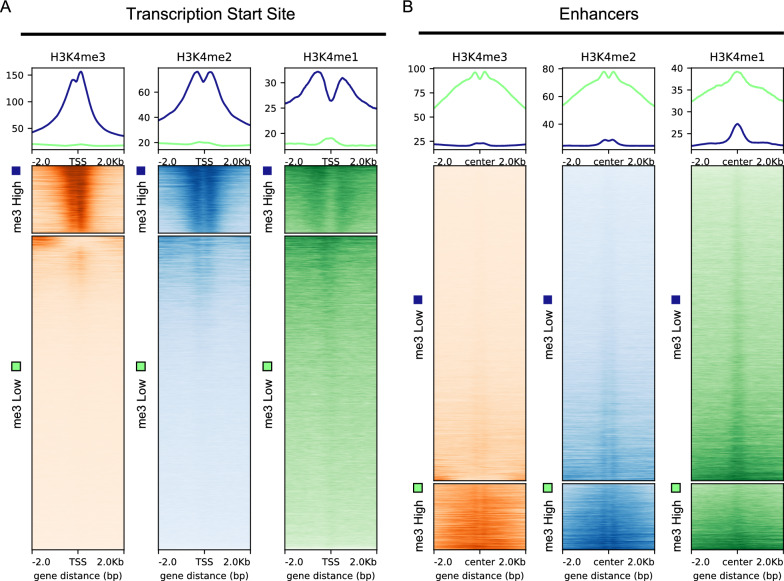
Spike-in normalised ChIP-seq profiles reveal characteristic H3K4 methylation patterns at transcription start sites and putative enhancers. **A** Wild Type embryos display expected H3K4 methylation architecture at protein-coding Transcription Start Sites (TSS). Genes were grouped into two clusters based on H3K4me3 levels (“me3 High” and “me3 Low”). H3K4me3 (red) display a double peak centred on the TSS, flanking H3K4me2 (blue) enrichment, and a valley of H3K4me1 (green) at the TSS with flanking enrichment. Aggregate plots (top) and heatmaps (bottom) show spike-in-normalised ChIP-seq signal centred on the TSS (± 2 kb). **B** Wild Type embryos display expected H3K4 methylation architecture at putative enhancers. Regions within the “me3 Low” cluster exhibit enhancer-like chromatin signatures, characterised by minimal H3K4me3 (red), moderate H3K4me2 (blue), and a strong central H3K4me1 peak (green). Aggregate plots (top) and heatmaps (bottom) show spike-in-normalised H3K4 methylation signal centred on enhancer midpoints (± 2 kb)

To assess the in vivo contributions of RBBP-5 and WDR-5 to H3K4 methylation, we performed spike-in-normalised ChIP-seq on *C. elegans* embryos for H3K4me1, H3K4me2, and H3K4me3. This approach enables accurate cross-genotype comparisons, especially important given the expected global reduction in H3K4 methylation in the *rbbp-5(-)* mutants. *Drosophila* chromatin and an antibody against the *Drosophila*-specific histone variant H2Av were used to control for technical variability during immunoprecipitation [64, 74]. The spike-in normalisation was critical for comparisons between the three genotypes analysed (see Suppl. Figure 1 for normalisation effects and Table S1 for scaling factors).To benchmark data quality, we compared our spike-in ChIP-seq datasets with publicly available modENCODE H3K4me1/2/3 data [63, 66]. Principal Component Analysis (PCA) demonstrated clustering of our samples alongside modENCODE replicates according to H3K4 methylation state (Suppl. Figure 2A), confirming reproducibility. Pearson correlation analysis further validated high concordance between our data and the modENCODE datasets (Suppl. Figure 2B). We next examined the genomic distribution of H3K4 methylation at transcription start sites (TSS) of protein-coding genes and putative active enhancers [67, 68]. Using k-means clustering, we segregated genes displaying high or low levels of H3K4me3. At TSS, genes with high H3K4me3 exhibited the expected “double-peak” profile accompanied by H3K4me1 depletion (Fig. 2A) [4, 63, 68]. Enhancers, in contrast, showed low H3K4me3 and relative enrichment in H3K4me1. Genes associated with low H3K4me3 displayed a characteristic H3K4me1 peak, consistent with previous studies (Fig. 2B) [63, 68]. Together, these analyses confirm that our spike-in ChIP-seq datasets are robust, accurately normalised, and comparable to the established modEncode reference data.

### RBBP-5 is required for bulk H3K4 methylation, whilst WDR-5 is critical for H3K4me3

**Fig. 3:**
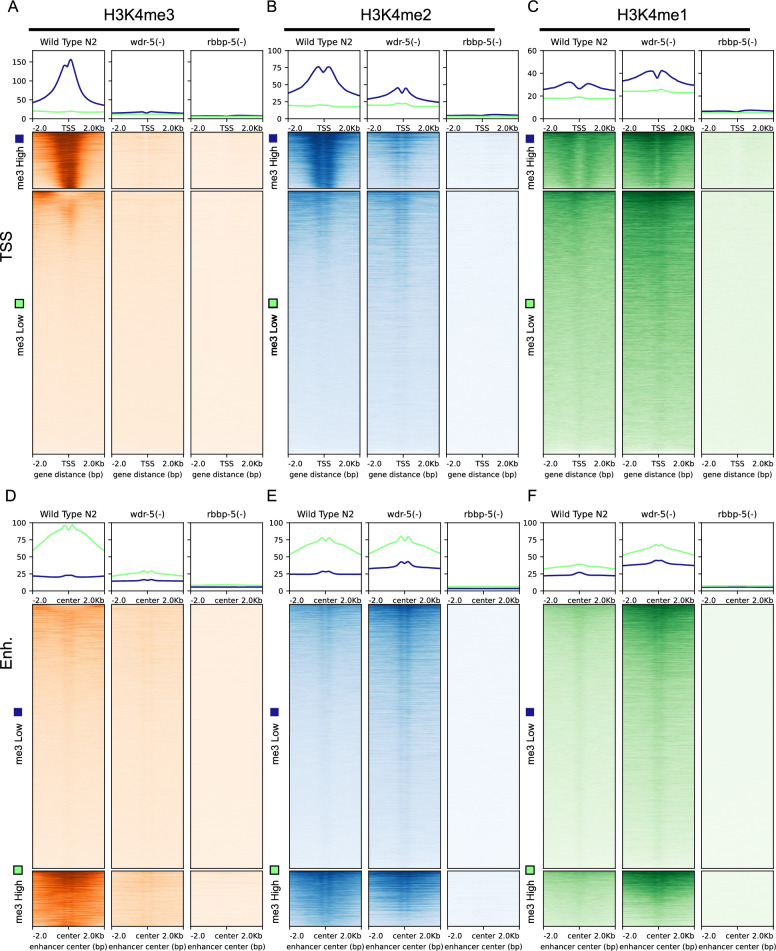
RBBP-5 is essential for deposition of all H3K4 methylation states, whereas WDR-5 is required for H3K4me3 but differentially modulates H3K4me2 and H3K4me1. **A**–**C** Heatmaps and aggregate plots (top) show spike-in-normalised H3K4me3/me2/me1 ChIP-seq signal centred on the TSS (± 2 kb) in wild-type (N2), *wdr-5(-)*, and *rbbp-5(-)* embryos. Genes were grouped into “me3 High” and “me3 Low” clusters based on wild-type H3K4me3 levels. **D**–**F** Heatmaps and aggregate plots show H3K4me3/me2/me1 ChIP-seq signal centred on enhancer midpoints (± 2 kb), again separated into “me3 Low” and “me3 High” clusters

To determine the relative contributions of WDR-5 and RBBP-5 to H3K4 methylation, we compared the deposition patterns of H3K4 mono- and multi-methylation between wild-type (N2) and the *wdr-5(-)* and *rbbp-5(-)* mutants using the same k-mean clustering approach described above (Fig. 2). In the absence of RBBP-5, H3K4me3 was almost completely lost at both TSS and enhancers (Fig. 3A and D). In *wdr-5(-)* embryos, H3K4me3 levels were also markedly reduced at TSS and enhancers, although the depletion was less severe than in the *rbbp-5(-)* mutant (Fig. 3A and D). For H3K4me2 deposition, loss of RBBP-5 resulted in a near-complete loss of signal at both TSS and enhancers (Fig. 3B and E). In contrast, the effects of WDR-5 loss on H3K4me2 were context-dependent: levels were reduced at TSS but increased at enhancers (Fig. 3B and E). Analysis of H3K4me1 levels revealed that RBBP-5 is essential for its deposition at both TSS and enhancers (Fig. 3C and F), whereas WDR-5 loss led to an accumulation of H3K4me1 at these features (Fig. 3C and F). This accumulation likely reflects impaired conversion to higher methylation states, leading to a build-up in H3K4me1. Overall, these results show that RBBP-5 is essential for all H3K4 methylation states, whilst WDR-5 is primarily required for H3K4me3 but exerts intriguing and opposite disruptions in H3K4me2 deposition.

### Loss of WDR-5 can cause differential disruptions in H3K4me2 deposition

**Fig. 4:**
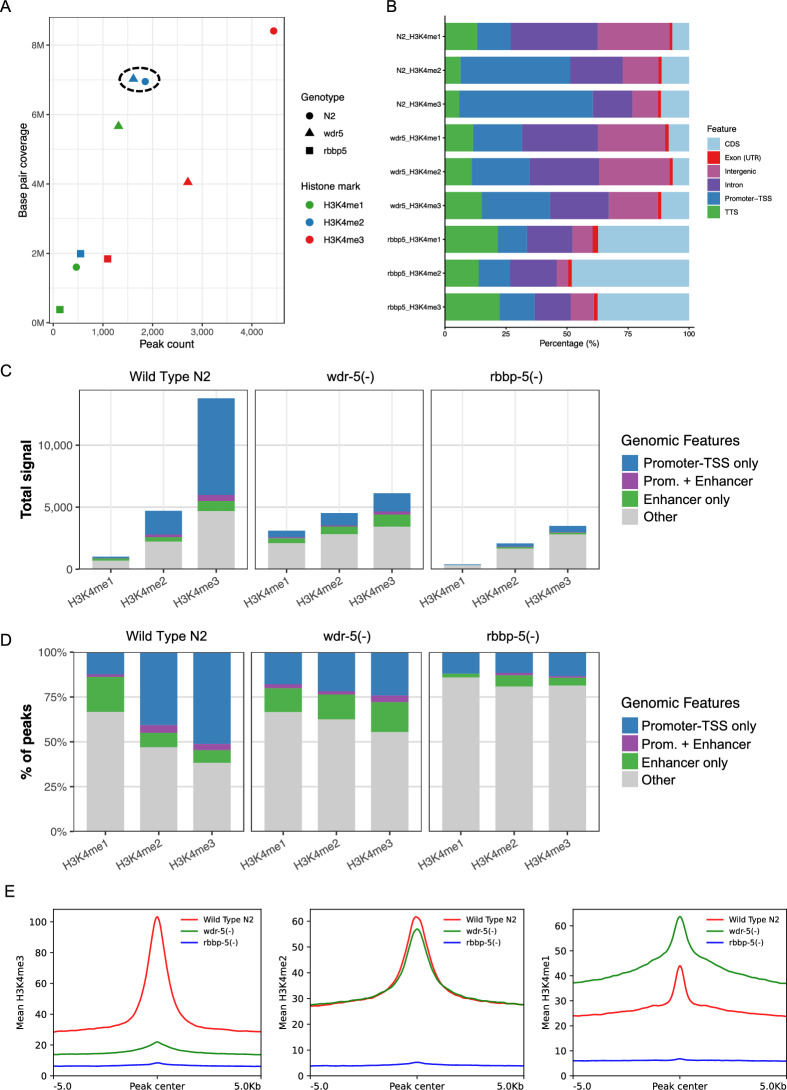
Peak-based analysis reveals context-dependent redistribution of H3K4me2 in the absence of WDR-5. **A** Scatterplot showing the relationship between peak count and total base-pair coverage for H3K4me1, H3K4me2, and H3K4me3 peaks in wild-type (N2), *wdr-5(-),* and *rbbp-5(-)* embryos. Each point represents a histone mark (colour-coded) for each genotype (shape-coded). The dotted circle highlights H3K4me2 for wild type and *wdr-5(-)* mutant. **B** Stacked barplots showing the percentage of peaks mapping to genomic features (CDS (coding sequence), Exon (UTR), intergenic regions, intron, promoter-TSS, and TTS (transcription termination site)). **C** Total H3K4me1/2/3 signal stratified by genomic feature. Barplots showing total ChIP-seq signal contributed by promoter-TSS, enhancer, and other features. **D** Relative distribution of H3K4me1/2/3 peaks across genomic features (% of peaks). **E** Line plots showing mean H3K4me3, H3K4me2, and H3K4me1 signal centred on peak summits (± 5 kb)

To further investigate the H3K4me2 alterations specific to the absence of WDR-5, we adopted a peak centric approach. We performed peak calling for H3K4me3/me2/me1 in wild type (N2), *wdr-5(-)*, and *rbbp-5(-)* mutants and analysed peaks based on their number, genomic coverage, signal intensity and association with genomic features. In the *rbbp-5(-)* mutant, both the number and coverage of peaks were strikingly reduced (Fig. 4A). By contrast, in the *wdr-5(-)* mutant, H3K4me2 peak number and coverage were nearly identical to wild type (Fig. 4A). We next examined peak distribution across six genomic features, including promoter-TSS, coding sequence (CDS), and intergenic regions. In wild type, approximately 55% and 45% of H3K4me3 and H3K4me2 peaks, respectively, localised to promoter-TSS features, whereas only ~ 14% of H3K4me1 peaks were found in this feature (Fig. 4B; Suppl. Table 2). In *wdr-5(-)* mutant, this differential enrichment was lost, with only ~ 28% and ~ 24% of H3K4me3 and H3K4me2 peaks, respectively, at promoter-TSS features. These results suggest that WDR-5 promotes the localisation of H3K4me2/me3 peaks to promoter-TSS features (Fig. 4B; Suppl. Table 2).

We then assessed peak signal intensity within promoter-TSS and enhancer regions. In wild type, roughly half of the total H3K4me2 signal originated from promoter-TSS features, with smaller contributions from enhancers and other genomic regions (Fig. 4C; Suppl. Table 2). In *wdr-5(-)* mutant, the promoter-TSS contribution decreased by approximately half, while enhancer-associated H3K4me2 signal increased proportionally. Additional increases from intergenic and intronic regions further compensated for this redistribution, resulting in total H3K4me2 levels that remained comparable to wild type (Fig. 4C; Suppl. Table 2). Analysis of peak percentages across the same genomic features revealed that loss of WDR-5 alters the relative distribution of H3K4 methylation states. For H3K4me1, the proportion of peaks mapping to enhancers decreased, whereas promoter-TSS-associated peaks increased relative to wild type. In contrast, both H3K4me2 and H3K4me3 peaks showed reduced representation at promoter-TSS and enhancer features in *wdr-5(-)* mutant. As expected, loss of RBBP-5 markedly reduced the percentage of peaks at these regulatory features for all three methylation states (Fig. 4D; Suppl. Table 2). To determine how individual peaks were affected, we compared mean signal intensities for H3K4me3, H3K4me2, and H3K4me1 across genotypes. H3K4me3 was severely depleted in both mutants (Fig. 4E), and RBBP-5 was essential for deposition of all three marks, consistent with earlier analyses (Fig. 3A–F). In contrast, H3K4me2 levels in *wdr-5(-)* mutant remained relatively high, showing only a modest global reduction (Fig. 4E). This outcome reflects reduced promoter-TSS signal coupled with increased enhancer and intergenic signal (Fig. 4C–D).

Independent validation by ChIP-qPCR, western blotting, and immunofluorescence largely supports these conclusions. Both *wdr-5(-)* and *rbbp-5(-)* mutants showed near-complete loss of H3K4me3 in all these assays (Suppl. Figures 3A–H and 4A). RBBP-5 was required for H3K4me2 deposition, whereas substantial H3K4me2 signal persisted in *wdr-5(-)* embryos, consistent with our ChIP-seq results and previous studies [49, 61], albeit not to the same extent (Suppl. Figures 3B, 4B, and 5). H3K4me1 levels were maintained at roughly wild type levels in *wdr-5(-)* embryos (Suppl. Figures 3C, 3G-H, and 4C), whereas the *rbbp-5(-)* mutant displayed markedly reduced levels (Suppl. Figures 3C and 4C), retaining 20–30% of wild type H3K4me1 signal by western blotting. This partial retention likely reflects methodological differences in sensitivity and assay context between genome-bound chromatin profiling (ChIP-seq/qPCR) versus bulk protein detection by western blotting. Interestingly, immunofluorescence revealed that WDR-5 is particularly critical early in embryogenesis but becomes partially dispensable later, as H3K4me2 levels recover. Together, these data indicate that although the main function of WDR-5 in embryos is to promote H3K4me3 deposition, its role in H3K4me2 regulation is complex, varying across genomic contexts and developmental stages. These findings underscore the distinct functions of WDR-5 and RBBP-5 in shaping the H3K4 methylation landscape.

### Chromosome X is differentially impacted by the absence of WDR-5 relative to autosomes

**Fig. 5:**
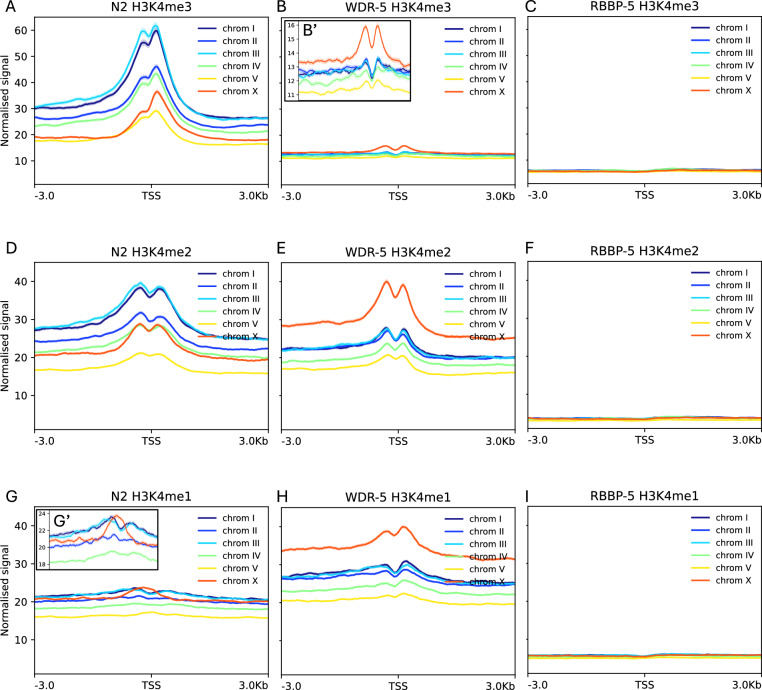
Chromosome X displays distinct H3K4 methylation responses to loss of WDR-5. Aggregate ChIP-seq signal plots show H3K4 methylation (± 3 kb from TSS) for each chromosome in wild-type (N2), *wdr-5(-)* and *rbbp-5(-)* embryos as indicated. **A** H3K4me3 displays a chromosomal hierarchy in wild-type embryos. **B** Loss of WDR-5 causes a reduction in H3K4me3 all chromosomes, but chromosome X is comparatively less affected than the autosomes (see B’ inset). **C** Loss of RBBP-5 nearly abolishes H3K4me3 across all chromosomes. **D** H3K4me2 levels in wild type shows a similar chromosomal hierarchy to H3K4me3. **E** Loss of WDR-5 causes a decrease in autosomal H3K4me2, whereas chromosome X shows a pronounced gain in signal. **F** Loss of RBBP-5 nearly abolishes H3K4me2 across all chromosomes. **G** H3K4me1 in wild-type embryos display a characteristic enrichment on chromosome X (see G’ inset). **H** Loss of WDR-5 increases H3K4me1 at all chromosomes, with the strongest elevation on chromosome X. **I** Loss of RBBP-5 nearly abolishes H3K4me1 across all chromosomes

To assess whether individual chromosomes are differentially affected by the absence of WDR-5, we compared H3K4 methylation at TSS across all six chromosomes. In wild type, the highest H3K4me3 levels were detected on chromosomes I and III, whereas chromosomes II and IV showed intermediate levels, and chromosomes X and V displayed the lowest levels (Fig. 5A). We next examined how this chromosomal hierarchy of H3K4me3 was altered in the *wdr-5(-)* mutant. As expected, H3K4me3 was globally reduced, but chromosome X was comparatively less affected than the autosomes (Fig. 5B). In contrast, the absence of RBBP-5 nearly abolished H3K4me3 deposition across all chromosomes (Fig. 5C). Analysis of H3K4me2 revealed a broadly similar chromosomal pattern: chromosomes I and III retained the highest levels, II and IV showed intermediate enrichment, and chromosome X exhibited a relative increase (Fig. 5D). In *wdr-5(-)* mutant, autosomal H3K4me2 levels declined, whereas chromosome X showed a pronounced gain in signal (Fig. 5E). As expected, only background levels were observed in the *rbbp-5(-)* mutant (Fig. 5F). For H3K4me1, wild type embryos displayed a characteristic enrichment on chromosome X not observed on autosomes (Fig. 5G). In the *wdr-5(-)* mutant, H3K4me1 levels increased genome-wide, with chromosome X showing the most prominent elevation (Fig. 5H). Only background signal remained in *rbbp-5(-)* embryos (Fig. 5I). Statistical assessment of input reads confirmed equivalent sequencing coverage across all chromosomes (Table S3). Together, these findings indicate that whilst WDR-5 is broadly required for H3K4 multi-methylation at TSS across the autosomes, chromosome X exhibits a distinct WDR-5 regulatory behaviour.

### WDR-5 has a greater impact on the transcriptome than RBBP-5

Given the distinct effects of WDR-5 and RBBP-5 on H3K4 methylation, we next asked how these differences would be reflected at the transcriptome level. To address this, we performed RNA-seq on staged embryos and identified differentially expressed genes (DEG) relative to wild type.

**Fig. 6:**
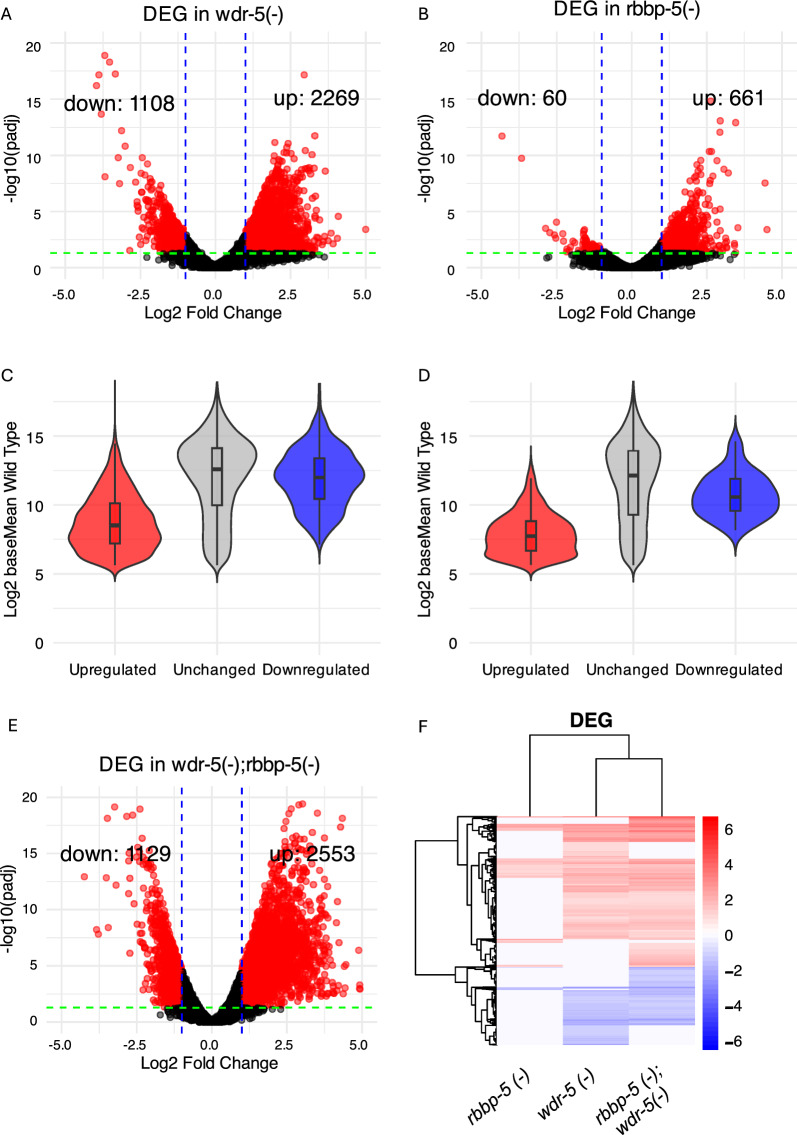
WDR-5 impacts the transcriptome more profoundly than RBBP-5. **A** and **B** Volcano plots showing differentially expressed genes (DEG) in *wdr-5*(-) and *rbbp-5*(-) mutants compared to wild-type. DEG (red dots) were defined as having > twofold change and adjusted *p* < 0.05. **C** and **D** Violin and box plots showing baseline (in wild-type) expression levels for upregulated, downregulated, and unchanged genes in each mutant. Downregulated genes have a statistically significant higher baseline expression in wild type than upregulated genes (p < 0.01; Mann–Whitney U test). **E** Volcano plot shows DEG in *rbbp-5(-); wdr-5(-)* embryos relative to wild type. **F** Hierarchical clustering reveals that the transcriptomes of the *wdr-5(-)* single mutant and the *rbbp-5(-); wdr-5(-)* double mutants are highly similar. Heatmap shows z-score normalised expression values for DEG across the *rbbp-5(-)* and *wdr-5(-)* single mutants and the double *rbbp-5(-); wdr-5(-)* mutant. Genes are clustered by expression pattern across the three genotypes

In *wdr-5(-)* embryos, we identified 3377 DEG, comprising 1108 downregulated genes and 2269 upregulated genes (Fig. 6A, Table S3). In contrast, the *rbbp-5(-)* mutant exhibited only 721 DEG, with 60 downregulated and 661 upregulated (Fig. 6B, Table S3). Quantitative RT-PCR on selected genes correlates with these RNA-seq data (Suppl. Figure 6). The predominance of upregulated genes was to an extent expected, even though H3K4 methylation is associated with active transcription, previous studies have also noted this effect [24, 59, 75]. Our results therefore align with transcriptomic analyses of catalytic-dead *set-2* and *set-16* mutants in young adults, and of *wdr-5(-)* and *set-2(-)* mutants in dissected gonads, which also showed a bias toward upregulation [59, 75]. However, a study analysing *set-2* mutants in early embryos reported a more balanced distribution [24]. To enable direct comparison with our analysis, we re-analysed this early embryonic dataset using our standard two-fold change cutoff and adjusted p-value (padj) < 0.05. We observed an enrichment of upregulated genes (587 upregulated versus 203 downregulated; Table S5), consistent with our results.

Because H3K4 methylation correlates with high transcriptional activity, we asked whether downregulated genes in the mutants correspond to highly expressed genes in wild type. To test this, we grouped DEG as downregulated, upregulated, or unchanged, and plotted their expression levels in wild type (baseMean) for each mutant (Fig. 6C–D). Downregulated genes in both mutants were significantly more highly expressed in wild type than upregulated genes (p < 0.01), indicating that expression level in wild type is associated with the direction of differential expression.

Finally, to quantify the overall transcriptomic impact of these mutants, we calculated a net directional change, which integrates both the direction (log2 fold change) and magnitude (baseMean) of expression changes. In *wdr-5(-)* embryos, this metric revealed a 28% net reduction in total transcript abundance, whereas *rbbp-5(-)* embryos showed a milder 9% reduction (Table S6). Thus, although both mutants exhibit more up- than downregulated genes, the overall transcript count decreases because the down regulation of highly expressed genes outweighs the upregulation of lowly expressed ones.

### WDR-5 exhibits RBBP-5-independent functions impacting on gene expression

We next asked whether the extensive transcriptomic changes observed in *wdr-5(-)* embryos could be attributed to the persistence of H3K4me1, H3K4me2, or residual H3K4me3. To test this, we generated a *rbbp-5(-); wdr-5(-)* double mutant, in which H3K4 methylation is expected to be abolished owing to the loss of RBBP-5.

We considered two possible outcomes. First, if the transcriptional changes in *wdr-5(-)* are primarily due to residual H3K4 methylation, the double mutant should resemble *rbbp-5(-)* (~ 700 DEG). Alternatively, if WDR-5 exerts functions independent of H3K4 methylation, the double mutant should more closely mirror the *wdr-5(-)* transcriptome (~ 3,000 DEG). It turns out that the *rbbp-5(-); wdr-5(-)* double mutant displayed 3682 DEG (Fig. 6E), a number comparable to the *wdr-5(-)* single mutant (3377 DEG; Fig. 6A). Of these, 712 genes, roughly 20%, showed restored wild type expression in the double mutant, indicating that persistent or residual H3K4 methylation of these DEG could have led to their mis-regulation. These 712 genes can be considered RBBP-5 dependent. Nevertheless, global analyses revealed that the overall transcriptomic profile of the *rbbp-5(-); wdr-5(-)* double mutant is more similar to *wdr-5(-)* than to *rbbp-5(-)*, indicating that those DEG are independent of RBBP-5. Clustering of DEG from single and double mutants confirmed that *wdr-5(-)* and the double mutant form a cluster together whereas *rbbp-5(-)* segregates separately (Fig. 6F). Principal component analysis (PCA), Euclidean distance, Pearson correlation, and hierarchical clustering of the full RNA-seq datasets yielded consistent results, again showing closer proximity between *wdr-5(-)* and *rbbp-5(-); wdr-5(-)* and identifying one divergent replicate (Suppl. Figure 7A-D). To determine whether the divergent rbbp-5_1 replicate biased our differential expression analysis, we examined replicate-level influence using Cook’s distance. Although the rbbp-5_1 replicate influenced a subset of genes (n = 277; Suppl. Figure 7E), the majority of these genes were not differentially expressed. Only seven genes with elevated Cook’s distance met our differential expression criteria. Importantly, relaxing those criteria identified only nine additional genes, indicating that replicate-specific variability neither masks nor inflates significantly the number of DEG identified in our RNA-seq analysis. Thus, the increased variability observed for *rbbp-5(-)* does not affect the conclusion that the *rbbp-5(-);wdr-5(-)* transcriptome is significantly closer to *wdr-5(-)* than to *rbbp-5(-)*. In addition, most DEG in *rbbp-5(-)* overlapped with those in both *wdr-5(-)* and the double mutant, consistent with RBBP-5 and WDR-5 functioning together within the SET/MLL complex [24, 76]. Two- and three-way Venn analyses confirmed highly significant overlaps (Suppl. Figure 8).

We then assessed whether DEG in the single and double mutants were differentially distributed between autosomes and the X chromosome, given that WDR-5 (but not RBBP-5) selectively affects H3K4 methylation on the X chromosome. Up- and downregulated genes in *wdr-5(-)* and *rbbp-5(-); wdr-5(-)* were, respectively, enriched and depleted on the X chromosome, further indicating that the transcriptome of *wdr-5(-)* closely resembles that of the double mutant (Suppl. Figure 9). Together, these data support a model by which WDR-5 contributes to gene regulation both through its role within the SET/MLL complex and via additional and RBBP-5-independent mechanisms during embryonic development.

### Gene ontology analysis on SET/MLL-linked and WDR-5-linked gene sets reveals overlapping and distinct functions

**Fig. 7:**
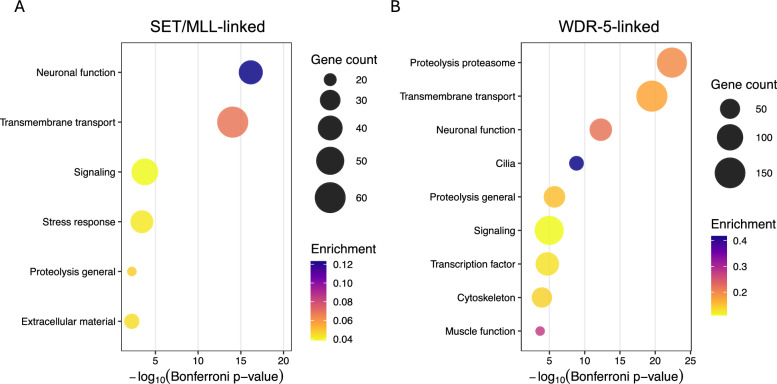
WormCat Gene Ontology analysis reveals both shared and distinct functional enrichments among SET/MLL-linked and WDR-5-linked gene sets. Bubble plots show enriched WormCat categories, displayed as -log_10_ (Bonferroni-adjusted p-value) on the *x-*axis. Bubble colour indicates the enrichment score (ratio of genes in the DEG set to genes annotated to that category), and bubble size reflects the number of differentially expressed genes (DEG) within each category. Categories are shown on the *y-*axis. **A** SET/MLL-linked DEG are defined, for convenience, as genes present in all three datasets: *rbbp-5(-)*, *wdr-5(-)*, and *rbbp-5(-); wdr-5(-)*. **B** WDR-5-linked DEG are defined, for convenience, as genes shared between the *wdr-5(-)* single mutant and the *rbbp-5(-); wdr-5(-)* double mutant, but absent from the *rbbp-5(-)* dataset

To explore the biological functions associated with SET/MLL-linked and WDR-5-linked activities, we performed Gene Ontology (GO) enrichment analysis using WormCat 2.0 [77]. We identified 570 DEG shared across all three genotypes (*wdr-5(-)*, *rbbp-5(-)*, and *rbbp-5(-); wdr-5(-)*). These DEG likely represent transcriptional outputs commonly impacted by SET/MLL complex dysfunction, although indirect effects cannot be excluded. We refer to these DEG as SET/MLL-linked and found enrichment for neuronal pathways, extracellular matrix components, transmembrane transport, proteolysis, signalling, and stress responses (Fig. 7A; Table S7). We then analysed the set of DEG shared only between the *wdr-5(-)* single and the *rbbp-5(-); wdr-5(-)* double mutants, which we interpret as candidates for WDR-5-linked functions that do not require SET/MLL-mediated H3K4 methylation because their misregulation in the absence of WDR-5 is not suppress by the loss of RBBP-5. This gene set also showed enrichment for neuronal, transmembrane transport, and signalling categories, indicating partial overlap with SET/MLL-linked functions. However, additional terms, including proteasome, cilia, transcription factors, cytoskeleton, and muscle, were uniquely represented in this WDR-5-linked set (Fig. 7B; Table S7). These specific GO terms may reflect pathways disproportionately affected by loss of WDR-5 only, i.e. not requiring RBBP-5, but indirect effects cannot be ruled out. Together, these analyses reveal that SET/MLL-linked and WDR-5-linked gene sets share functional signatures in neuronal, transmembrane transport, and signalling processes, while WDR-5 appears to influence distinct pathways (including proteasome, ciliary, and muscle-associated genes) that are not captured within the shared SET/MLL-linked transcriptome.

## Discussion

Our study reveals that WDR-5 and RBBP-5, two conserved scaffolding components of the SET/MLL H3K4 methyltransferase complex, contribute distinctly to H3K4 methylation and gene regulation during *C. elegans* embryogenesis. Using spike-in-normalised ChIP-seq, we show that RBBP-5 is broadly required for H3K4 mono-, di-, and tri-methylation, whereas WDR-5 is primarily required for H3K4me3 and exerts nuanced and feature-specific effects on H3K4me2. These findings are consistent with structural studies in metazoans showing that RBBP5 forms a central scaffold engaging both nucleosomal DNA and histone surfaces to stabilise and activate the catalytic module, while WDR5 fine-tunes complex assembly and activity in an MLL-paralogue-dependent manner [36, 78–80]. Our findings are also broadly consistent with prior work in *C. elegans* embryos [51, 52, 81], while extending these observations by providing quantitative, genome-wide comparisons across methylation states and genomic features.

A striking outcome of our analysis is that WDR-5 has far broader transcriptional consequences than RBBP-5, even though RBBP-5 is required for nearly all H3K4 methylation. The magnitude of transcriptomic disruption in *wdr-5(-)* embryos, more than four-fold greater than in *rbbp-5(-)*, suggests that many WDR-5-linked gene expression changes occur independently of RBBP-5 and by extension H3K4 methylation. This interpretation is supported by the *rbbp-5(-); wdr-5(-)* double mutant, where the majority of *wdr-5(-)* specific transcriptional changes persist despite the near-complete loss of H3K4me1/2/3. These results therefore motivated us to put forward a speculative model by which WDR-5 performs functions in parallel to H3K4 methylation to regulate embryonic transcription and therefore act as a multi-functional hub in the nucleus as suggested by Guarnaccia et al. in vertebrates [40].

WDR5 was first characterised in mammals as a core component of the H3K4 methylation complex [82]. However, it was later found in other complexes notably the NSL and ATAC histone acetyltransferase complexes [43, 83]. WDR5 can also be found in histone deacetylases complexes such as RPD3 HDAC in yeast (or mSin3a-HDAC1 in mammals) and the NuRD complex [46]. ING2, a component of the sSin3a-HDAC1, interacts via its PHD domain with H3K4me3 to stimulate deacetylation and repress transcription [84]. It was also shown that H3K4me2/me3 can recruit RPD3 HDAC to repress cryptic transcription [29]. In *C. elegans*, WDR-5 is a component of the orthologous Sin3S HDAC repressive complex containing HDA-1 [24]. Because histone deacetylases are classically linked to transcriptional repression, this interaction raises the possibility that WDR-5 may contribute to HDAC-dependent gene silencing independently of its role in H3K4 methylation. Consistent with such a model, we observed that the majority of differentially expressed genes in *wdr-5(-)* embryos were upregulated (67%), suggesting a bias toward transcriptional de-repression. This expression pattern is compatible with a potential role of WDR-5 in repressive chromatin regulation. To further explore this possibility, we sought suitable RNA-seq datasets from HDAC pathway mutants. However, the available *hda-1(RNAi)* and *sin-3* mutant embryo datasets were generated at developmental stages that differ from ours, being either earlier or later than those analysed in this study. Given the highly dynamic nature of embryonic transcription, such stage differences substantially limit direct comparisons. Indeed, we did not detect statistically significant overlap between upregulated genes in *wdr-5(-)* embryos and *hda-1* or *sin-3* datasets [24, 85]. Thus, our data neither conclusively support nor refute a direct role for WDR-5 within HDAC-mediated repression during the embryonic window analysed here.

Nevertheless, work in other species still supports the existence of H3K4 methylation-independent functions for WDR5. For example, WDR5 point mutations defective in H3K4 methylation could still rescue specific phenotypes such as mouse embryonic stem cells self-renewal defects and de-repression of germ cell specific genes [48] as well as left–right patterning of the heart in Xenopus [86], indicating that H3K4 methylation-independent activity is conserved in vertebrates. Thus, it is plausible that WDR-5 could act as a multi-functional hub regulating both activation and context-dependent repression of transcription.

RBBP-5 is essential to the deposition of H3K4 methyl groups and appears more specialised towards H3K4 methylation. A study investigating crosstalk between the NSL and SET/MLL complexes in *Drosophila* has shown that depletion of RBBP5 affects H3K4me2 deposition but not H4K16 acetylation. These results indicate that RBBP5 is not directly affecting histone acetylation, whereas WDR5 inactivation can affect both H3K4 methylation and H4K16 acetylation [44]. In addition, a *C. elegans* study investigating masculinisation of the germline found that this phenotype arises in the absence of WDR-5 but not in the absence of RBBP-5, indicating that WDR-5 has H3K4 methylation-independent activity but not RBBP-5 [87]. Interestingly, loss-of-function mutations in human RBBP-5 have recently been associated with neurodevelopmental disorder, short stature and microcephaly [88], highlighting the importance of H3K4 methylation for neuronal function.

Our analysis has also revealed a chromosomal hierarchy in H3K4me3 enrichment at transcription start sites, which closely mirrors the pattern of phenotypic enrichment observed through systematic RNAi screening [89]. Specifically, chromosomes I and III exhibit the highest levels of H3K4me3, followed by chromosomes II and IV, and with chromosomes V and X showing the lowest enrichment. This pattern is consistent with previous findings showing that chromosomes I and III are relatively enriched for active chromatin marks, including H3K4 methylation, whereas chromosomes V and X are comparatively depleted [63]. One possible explanation linking H3K4me3 enrichment to RNAi phenotypic outcomes is that genes which are robustly transcribed (and therefore more heavily marked by H3K4me3) are more likely to yield observable phenotypes when knocked down by RNAi. Although the mechanisms coordinating transcriptional regulation at the chromosome-wide level remain poorly understood, it is plausible that differential H3K4me3 enrichment reflects the partitioning of chromosomes into distinct chromatin environments during early embryogenesis. Higher levels of H3K4me3 on chromosomes I and III may indicate a more open, transcriptionally permissive architecture, possibly maintained through large-scale domain organisation or preferential spatial positioning within the nucleus. Conversely, the lower H3K4me3 levels on chromosomes V and X could reflect sequestration into less active chromatin territories. Notably, data from a recent *C. elegans* study investigating partitioning of chromatin states in the germlines has also revealed that transcriptionally active domains (marked by high H3K4me3 and H3K36me3) follow a similar chromosomal hierarchy [90]. These chromosome-wide biases in chromatin accessibility and transcriptional competence may underlie the observed correspondence between H3K4me3 and RNAi phenotype enrichment.

Our work has limitations. We cannot formally exclude the possibility that other WDR-5 paralogues (WDR-5.2, WDR-5.3) compensate for loss of WDR-5, although biochemical studies have not detected these paralogues in the SET/MLL complex [24, 76]. Even though previous analysis has shown that these paralogues do not appear to carry out H3K4 methylation activity in germlines or early embryos [87], it remains a possibility that compensation by replacement could occur at specific or later stages during embryogenesis. Indeed, immuno-fluorescence revealed that WDR-5 is essential for early deposition of H3K4me2 but appears to be partially dispensable later during development. This observation could, at least in part, explain some apparent discrepancies regards to the role of WDR-5 in deposition of H3K4me2. However, other factors, especially when using different techniques with different fixation protocols, for example, are also quite possibly contributing to these differences between studies [49, 51, 61]. Nevertheless, the substantial levels of H3K4me2 detected make it plausible that the absence of WDR-5 is somehow compensated by either paralogues or the recruitment of additional H3K4 methyltransferases [91]. However, because the transcriptome of the double *rbbp-5(-);wdr-5(-)* mutant remains strongly WDR-5-like despite the absence of H3K4 methylation, this putative compensatory activity that could generate a somewhat erroneous SET/MLL complex would still be independent of RBBP-5.

In addition, we cannot formally exclude the possibility that a uniform reduction in transcriptional output, or a disproportionate reduction in highly expressed genes enriched for H3K4 methylation, could introduce a normalisation bias in the RNA-seq analyses of *rbbp-5(-)* and *wdr-5(-)* mutants. In such a scenario, changes in highly abundant transcripts could influence scaling during normalisation, thereby affecting the relative representation of lower-abundance genes. Thus, while our analyses reveal robust and reproducible differences in relative gene expression, they do not directly measure absolute transcription rates. However, a severe global reduction in transcription is difficult to reconcile with the biological phenotypes observed in *wdr-5(-)* animals. Under the conditions used in our study, the *wdr-5(-)* embryos are viable, develop normally, and give rise to fertile adults, features that argue against a severe and system-wide reduction in transcription. This interpretation is also coherent with studies showing that H3K4 methylation contribution to basal transcription is not vital [92, 93]. For example, in *Drosophila*, Hödl and Basler replaced all 46 canonical His3.2 copies with non-methylatable H3K4A/R variants and deleted both H3.3 loci, thereby eliminating H3K4 methylation from all H3 species. Despite the absence of detectable H3K4me3, mutant cells were able to proliferate, differentiate, and activate developmental target genes, albeit with reduced growth rates [94]. In contrast, more recent studies have shown that acute loss of H3K4me3 reduces pause-release during elongation, a regulatory step important for stress responses, cell fate transition and signalling pathways [18, 20], which suggests its role may be more prominent in dynamic rather than steady-state transcription. Future studies using acute degradation systems combined with nascent transcription assays, e.g. GRO-seq, and RNA polymerase II occupancy profiling will be required to directly address this point.

We also observe that the X chromosome displays elevated H3K4me1 relative to autosomes, a feature exaggerated in *wdr-5(-)* embryos. Although speculative, these patterns raise the possibility that H3K4me1 contributes to transcriptional dampening on chromosome X. But perhaps as shown during the early stages of X chromosome inactivation in mammals [95], elevated H3K4me1 levels may reflect reduced transcription and the incomplete conversion into H3K4me2 and H3K4me3. Additionally, components of the SET/MLL complex (such as DPY-30) also localise to X chromosomal regions associated with dosage compensation [96]. Although ASH-2 depletion does not disrupt DC recruitment, the co-localisation of SET/MLL and DC machinery could influence local methylation dynamics by preventing enrichment of H3K4me3 and thereby increasing the levels of H3K4me1.

In conclusion**,** our study shows that WDR-5 and RBBP-5 exert distinct and separable functions in regulating the embryonic chromatin landscape. WDR-5 acts as a facilitator of H3K4me3 deposition and possibly as a multifunctional chromatin regulator whose influence on gene expression is RBBP-5 independent and thus likely extends beyond the SET/MLL methylation pathway. RBBP-5, by contrast, plays a central catalytic role within the SET/MLL complex for establishing all H3K4 methylation states. Future work aimed at defining the full embryonic interactome of WDR-5 in vivo and dissecting the temporal integration of its activities across complexes will be critical for understanding how chromatin regulatory hubs coordinate developmental gene expression programs.

## Supplementary Information

Supplementary Material 1: Figure 1. Effects of normalisation on H3K4 methylation profiles. A Heatmaps showing histone H3K4 methylation ChIP-seq signal intensities centred on TSS (±2 kb) in wild-type (N2), wdr-5(-), and rbbp-5(-) mutants. For each genotype pre-correction (raw) and post-correction (normalised) ChIP-seq signals are shown for H3K4me3, me2, and me1.

Supplementary Material 2: Figure 2. Benchmarking spike-in ChIP-seq datasets against modENCODE using PCA and Pearson correlation analysis. A Principal Component Analysis (PCA) of usable ChIP-seq tags from this study (N2) and public C. elegans datasets from modENCODE (R1, R2) for H3K4me1, H3K4me2, and H3K4me3. Samples cluster primarily by histone modification (color-coded) and by replicate, indicating high signal specificity and low technical noise. B Pearson correlation matrix of usable ChIP-seq tags shows high intra-group correlation, especially among replicates targeting the same modification. Hierarchical clustering groups samples by H3K4me1, H3K4me2, and H3K4me3, with datasets from this study (N2) and modENCODE (R1, R2) generally in strong agreement.

Supplementary Material 3: Figure 3. ChIP-qPCR with associated track and Western blots showing broad agreement with the spike-in-normalised ChIP-seq data. (A-C) ChIP-qPCR at three loci (an untranscribed region, act-1 and ftt-2) for H3K4me3/me2/me1 into wild type (WT) embryos, wdr-5(-) and rbbp-5(-) mutants. (D-F) Associated track from the spike-in-normalised ChIP-seq data. H3K4me3/me2/me1 are colour coded in red, blue and green respectively and genotype indicated on the left. The black box indicates approximate location of the primer pairs (see Table S8 for exact location) and the top-left values within square brackets are signal levels selection. (G-H) Western blots on mixed stage embryos from wild type (N2), wdr-5(-) and rbbp-5(-) mutants for H3K4me3/me2/me1 normalised against H3 and performed three times independently. Error bars are S.E.M.

Supplementary Material 4: Figure 4. Immuno-fluorescence staining against H3K4me3/me2/me1 and DAPI staining for the indicated genotype has been performed on embryos from the P1 stage (two-cell) to Z2-Z3 showing that loss of WDR-5 is strikingly preventing H3K4me2 deposition at early stages of embryogenesis, but only partially at late stages of embryogenesis. (A-C) Photographs of representative immuno-fluorescence staining at P4 and Z2-Z3. Scale bar is 10μm and indicated to the bottom right. (D-F) The abundance of each mark in the indicated genotype and developmental stages have been calculated from quantifying 6 to 8 cells for each stage and normalising the H3K4 methylation signal by DAPI signal intensity.

Supplementary Material 5: Figure 5. IGV tracks from spike-in-normalised ChIP-seq as well as modEncode data. (A-B) Comparisons between this study ChIP-seq data and modENCODE data as well as showing the effects that loss of WDR-5 and RBBP-5 have on H3K4me3/me2/me1 at a TSS and an enhancer at loci indicated at the top left. The box indicate the approximate location of the TSS or enhancer as defined in our study.

Supplementary Material 6: Figure 6. Quantitative RT-PCR in wdr-5(-) and rbbp-5(-) mutants on selected downregulated, upregulated genes as well as on genes displaying unchanged levels of expression correlate with RNA-seq data. Experiments were performed in triplicates on separate biological samples and normalised using act-1 and the ΔΔct method. The error bars represent the ±SEM.

Supplementary Material 7: Figure 7. Analysis of RNA-seq data showing that the transcriptome of the single wdr-5(-) mutant is closer to the double rbbp-5(-);wdr-5(-) mutant. (A) PCA shows that most samples cluster according to their respective genotype except for replicate rbbp-5(-)_1. (B) Euclidean distances show that the single wdr-5(-) mutant is closer to the double rbbp-5(-);wdr-5(-) mutant when compared with the distance between the single rbbp-5(-) mutant and the double rbbp-5(-);wdr-5(-) mutant, (17 versus 45.29). (C) Pearson correlation shows that wdr-5(-) and the double rbbp-5(-);wdr-5(-) form a distinct cluster whereas wild type and two replicates of rbbp-5(-) another. (D) Hierarchical clustering shows that wdr-5(-) and the double rbbp-5(-);wdr-5(-) form a distinct cluster whereas wild type and two replicates of rbbp-5(-) another. (E) Cook’s analysis demonstrating the limited influence of the rbbp-5_1 replicate on differential expression results. After expression filtering, 277 Cook’s-sensitive genes (Cook’s >1) were retained. Of these, only 7 met our standard differential expression criteria, and a total of 16 met relatively more relaxed criteria. Points are coloured according to smoothed gene density (two-dimensional kernel density estimate). Dashed and dotted lines indicate relaxed (|log2FC > 0.75 and p-adj < 0.1) as well as thresholds used throughout our study (|log2FC >1| and p-adj < 0.05), respectively.

Supplementary Material 8: Figure 8. Venn diagrams (2-way and 3-way) showing that WDR-5 and RBBP-5 are part of the SET/MLL complex. (A-C) 2-way Venn diagrams showing the overlap of mis-, up- and downregulated genes between the rbbp-5(-) and wdr-5(-) single mutants. (D-F) 3-way Venn diagrams showing the overlap of mis-, up- and downregulated between rbbp-5(-), wdr-5(-), and rbbp-5(-); wdr-5(-) mutants.

Supplementary Material 9: Figure 9. Enrichment plots for chromosomal analysis of RNA-deq data showing that DEG on chromosome X are over-represented for upregulated genes but under-represented for downregulated genes in the single wdr-5(-) and double rbbp-5(-);wdr-5(-) mutants, but not in rbbp-5(-). For each mutant and gene-expression direction (up or down), log2 (observed/expected) enrichment values were plotted on the x-axis, with chromosomes (I–V, X) on the y-axis. Error bars represent 95 % confidence intervals of the observed proportion. Vertical dashed lines indicate the random expectation (0) and dotted lines mark the ±20 % biological threshold (±log2 1.2 ≈ ±0.26). Points were coloured red when both criteria (FDR ≤ 0.01 and ≥ 20 % enrichment) were satisfied, and grey otherwise. (A-B) Forest plots style for up- or downregulated genes (wdr-5(-) DEG up or wdr-5(-) DEG down ) for each chromosome (C-D) Forest plots style for up- or downregulated genes (rbbp-5(-) DEG up or rbbp-5(-) DEG down ) for each chromosome. (E-F) Forest plots style for up- or downregulated genes (rbbp-5(-);wdr-5(-) DEG up or rbbp-5(-);wdr-5(-) DEG down ) for each chromosome.

Supplementary Material 10.

Supplementary Material 11.

Supplementary Material 12.

Supplementary Material 13.

Supplementary Material 14.

Supplementary Material 15.

Supplementary Material 16.

Supplementary Material 17.

## Data Availability

The ChIP-seq data have been deposited in the GEO repository under ID code GSE94639 and RNA-seq data have been deposited in the ArrayExpress repository under ID code E-MTAB-15080.

## References

[1] Palacio M, Taatjes DJ. Merging established mechanisms with new insights: condensates, hubs, and the regulation of RNA polymerase II transcription. J Mol Biol. 2022;434:167216. 34474085 10.1016/j.jmb.2021.167216 PMC8748285

[2] Bannister AJ, Kouzarides T. Regulation of chromatin by histone modifications. Cell Res. 2011;21:381–95. 21321607 10.1038/cr.2011.22 PMC3193420

[3] Kouzarides T. Chromatin modifications and their function. Cell. 2007;128:693–705. 17320507 10.1016/j.cell.2007.02.005

[4] Wang H, Helin K. Roles of H3K4 methylation in biology and disease. Trends Cell Biol. 2024;35(2):115–28. 38909006 10.1016/j.tcb.2024.06.001

[5] Chong SY, Cutler S, Lin JJ, Tsai CH, Tsai HK, Biggins S, et al. H3K4 methylation at active genes mitigates transcription-replication conflicts during replication stress. Nat Commun. 2020;11:809. 32041946 10.1038/s41467-020-14595-4 PMC7010754

[6] Hsu PL, Li H, Lau HT, Leonen C, Dhall A, Ong SE, et al. Crystal structure of the COMPASS H3K4 methyltransferase catalytic module. Cell. 2018;174(1106–1116):e1109. 10.1016/j.cell.2018.06.038 PMC6108940 30100181

[7] Schibler A, Koutelou E, Tomida J, Wilson-Pham M, Wang L, Lu Y, et al. Histone H3K4 methylation regulates deactivation of the spindle assembly checkpoint through direct binding of Mad2. Genes Dev. 2016;30:1187–97. 27198228 10.1101/gad.278887.116 PMC4888839

[8] Wang S, Meyer DH, Schumacher B. H3K4me2 regulates the recovery of protein biosynthesis and homeostasis following DNA damage. Nat Struct Mol Biol. 2020;27:1165–77. 33046905 10.1038/s41594-020-00513-1

[9] Bieberstein NI, Carrillo Oesterreich F, Straube K, Neugebauer KM. First exon length controls active chromatin signatures and transcription. Cell Rep. 2012;2:62–8. 22840397 10.1016/j.celrep.2012.05.019

[10] Sims RJ 3rd, Millhouse S, Chen CF, Lewis BA, Erdjument-Bromage H, Tempst P, et al. Recognition of trimethylated histone H3 lysine 4 facilitates the recruitment of transcription postinitiation factors and pre-mRNA splicing. Mol Cell. 2007;28:665–76. 18042460 10.1016/j.molcel.2007.11.010 PMC2276655

[11] Barski A, Cuddapah S, Cui K, Roh TY, Schones DE, Wang Z, et al. High-resolution profiling of histone methylations in the human genome. Cell. 2007;129:823–37. 17512414 10.1016/j.cell.2007.05.009

[12] Guenther MG, Levine SS, Boyer LA, Jaenisch R, Young RA. A chromatin landmark and transcription initiation at most promoters in human cells. Cell. 2007;130:77–88. 17632057 10.1016/j.cell.2007.05.042 PMC3200295

[13] Schneider R, Bannister AJ, Myers FA, Thorne AW, Crane-Robinson C, Kouzarides T. Histone H3 lysine 4 methylation patterns in higher eukaryotic genes. Nat Cell Biol. 2004;6:73–7. 14661024 10.1038/ncb1076

[14] Lauberth SM, Nakayama T, Wu X, Ferris AL, Tang Z, Hughes SH, et al. H3K4me3 interactions with TAF3 regulate preinitiation complex assembly and selective gene activation. Cell. 2013;152:1021–36. 23452851 10.1016/j.cell.2013.01.052 PMC3588593

[15] Li H, Ilin S, Wang W, Duncan EM, Wysocka J, Allis CD, et al. Molecular basis for site-specific read-out of histone H3K4me3 by the BPTF PHD finger of NURF. Nature. 2006;442:91–5. 16728978 10.1038/nature04802 PMC4690523

[16] Vermeulen M, Eberl HC, Matarese F, Marks H, Denissov S, Butter F, et al. Quantitative interaction proteomics and genome-wide profiling of epigenetic histone marks and their readers. Cell. 2010;142:967–80. 20850016 10.1016/j.cell.2010.08.020

[17] Vermeulen M, Mulder KW, Denissov S, Pijnappel WW, van Schaik FM, Varier RA, et al. Selective anchoring of TFIID to nucleosomes by trimethylation of histone H3 lysine 4. Cell. 2007;131:58–69. 17884155 10.1016/j.cell.2007.08.016

[18] Hu S, Song A, Peng L, Tang N, Qiao Z, Wang Z, et al. H3K4me2/3 modulate the stability of RNA polymerase II pausing. Cell Res. 2023;33:403–6. 36922644 10.1038/s41422-023-00794-3 PMC10156655

[19] Santos-Rosa H, Schneider R, Bannister AJ, Sherriff J, Bernstein BE, Emre NC, et al. Active genes are tri-methylated at K4 of histone H3. Nature. 2002;419:407–11. 12353038 10.1038/nature01080

[20] Wang H, Fan Z, Shliaha PV, Miele M, Hendrickson RC, Jiang X, et al. H3K4me3 regulates RNA polymerase II promoter-proximal pause-release. Nature. 2023;615:339–48. 36859550 10.1038/s41586-023-05780-8 PMC9995272

[21] Benayoun BA, Pollina EA, Ucar D, Mahmoudi S, Karra K, Wong ED, et al. H3K4me3 breadth is linked to cell identity and transcriptional consistency. Cell. 2014;158:673–88. 25083876 10.1016/j.cell.2014.06.027 PMC4137894

[22] Chen K, Chen Z, Wu D, Zhang L, Lin X, Su J, et al. Broad H3K4me3 is associated with increased transcription elongation and enhancer activity at tumor-suppressor genes. Nat Genet. 2015;47:1149–57. 26301496 10.1038/ng.3385 PMC4780747

[23] Hughes AL, Kelley JR, Klose RJ. Understanding the interplay between CpG island-associated gene promoters and H3K4 methylation. Biochim Biophys Acta Gene Regul Mech. 2020;1863:194567. 32360393 10.1016/j.bbagrm.2020.194567 PMC7294231

[24] Beurton F, Stempor P, Caron M, Appert A, Dong Y, Chen RA, et al. Physical and functional interaction between SET1/COMPASS complex component CFP-1 and a Sin3S HDAC complex in C. elegans. Nucleic Acids Res. 2019;47:11164–80. 31602465 10.1093/nar/gkz880 PMC6868398

[25] Bernstein BE, Mikkelsen TS, Xie X, Kamal M, Huebert DJ, Cuff J, et al. A bivalent chromatin structure marks key developmental genes in embryonic stem cells. Cell. 2006;125:315–26. 16630819 10.1016/j.cell.2006.02.041

[26] Rinn JL, Kertesz M, Wang JK, Squazzo SL, Xu X, Brugmann SA, et al. Functional demarcation of active and silent chromatin domains in human HOX loci by noncoding RNAs. Cell. 2007;129:1311–23. 17604720 10.1016/j.cell.2007.05.022 PMC2084369

[27] Briggs SD, Bryk M, Strahl BD, Cheung WL, Davie JK, Dent SY, et al. Histone H3 lysine 4 methylation is mediated by Set1 and required for cell growth and rDNA silencing in *Saccharomyces cerevisiae*. Genes Dev. 2001;15:3286–95. 11751634 10.1101/gad.940201 PMC312847

[28] Kim T, Buratowski S. Dimethylation of H3K4 by Set1 recruits the Set3 histone deacetylase complex to 5’ transcribed regions. Cell. 2009;137:259–72. 19379692 10.1016/j.cell.2009.02.045 PMC2802783

[29] Pinskaya M, Gourvennec S, Morillon A. H3 lysine 4 di- and tri-methylation deposited by cryptic transcription attenuates promoter activation. EMBO J. 2009;28:1697–707. 19407817 10.1038/emboj.2009.108 PMC2699354

[30] Ryu HY. Histone modification pathways suppressing cryptic transcription. Epigenomes. 2024. 10.3390/epigenomes8040042. 39584965 10.3390/epigenomes8040042 PMC11586988

[31] Creyghton MP, Cheng AW, Welstead GG, Kooistra T, Carey BW, Steine EJ, et al. Histone H3K27ac separates active from poised enhancers and predicts developmental state. Proc Natl Acad Sci U S A. 2010;107:21931–6. 21106759 10.1073/pnas.1016071107 PMC3003124

[32] Heintzman ND, Hon GC, Hawkins RD, Kheradpour P, Stark A, Harp LF, et al. Histone modifications at human enhancers reflect global cell-type-specific gene expression. Nature. 2009;459:108–12. 19295514 10.1038/nature07829 PMC2910248

[33] Miller T, Krogan NJ, Dover J, Erdjument-Bromage H, Tempst P, Johnston M, et al. COMPASS: a complex of proteins associated with a trithorax-related SET domain protein. Proc Natl Acad Sci U S A. 2001;98:12902–7. 11687631 10.1073/pnas.231473398 PMC60797

[34] Nagy PL, Griesenbeck J, Kornberg RD, Cleary ML. A Trithorax-group complex purified from *Saccharomyces cerevisiae* is required for methylation of histone H3. Proc Natl Acad Sci U S A. 2002;99:90–4. 11752412 10.1073/pnas.221596698 PMC117519

[35] Roguev A, Schaft D, Shevchenko A, Pijnappel WW, Wilm M, Aasland R, et al. The *Saccharomyces cerevisiae* Set1 complex includes an Ash2 homologue and methylates histone 3 lysine 4. EMBO J. 2001;20:7137–48. 11742990 10.1093/emboj/20.24.7137 PMC125774

[36] Xue H, Yao T, Cao M, Zhu G, Li Y, Yuan G, et al. Structural basis of nucleosome recognition and modification by MLL methyltransferases. Nature. 2019;573:445–9. 31485071 10.1038/s41586-019-1528-1

[37] Shilatifard A. The COMPASS family of histone H3K4 methylases: mechanisms of regulation in development and disease pathogenesis. Annu Rev Biochem. 2012;81:65–95. 22663077 10.1146/annurev-biochem-051710-134100 PMC4010150

[38] Wenzel D, Palladino F, Jedrusik-Bode M. Epigenetics in C. elegans: facts and challenges. Genesis. 2011;49:647–61. 21538806 10.1002/dvg.20762

[39] Sze CC, Shilatifard A. MLL3/MLL4/COMPASS family on epigenetic regulation of enhancer function and cancer. Cold Spring Harb Perspect Med. 2016. 10.1101/cshperspect.a026427. 27638352 10.1101/cshperspect.a026427 PMC5088509

[40] Guarnaccia AD, Tansey WP. Moonlighting with WDR5: a cellular multitasker. J Clin Med. 2018. 10.3390/jcm7020021. 29385767 10.3390/jcm7020021 PMC5852437

[41] Dou Y, Milne TA, Tackett AJ, Smith ER, Fukuda A, Wysocka J, et al. Physical association and coordinate function of the H3 K4 methyltransferase MLL1 and the H4 K16 acetyltransferase MOF. Cell. 2005;121:873–85. 15960975 10.1016/j.cell.2005.04.031

[42] Mendjan S, Taipale M, Kind J, Holz H, Gebhardt P, Schelder M, et al. Nuclear pore components are involved in the transcriptional regulation of dosage compensation in *Drosophila*. Mol Cell. 2006;21:811–23. 16543150 10.1016/j.molcel.2006.02.007

[43] Dias J, Van Nguyen N, Georgiev P, Gaub A, Brettschneider J, Cusack S, et al. Structural analysis of the KANSL1/WDR5/KANSL2 complex reveals that WDR5 is required for efficient assembly and chromatin targeting of the NSL complex. Genes Dev. 2014;28:929–42. 24788516 10.1101/gad.240200.114 PMC4018492

[44] Zhao X, Su J, Wang F, Liu D, Ding J, Yang Y, et al. Crosstalk between NSL histone acetyltransferase and MLL/SET complexes: NSL complex functions in promoting histone H3K4 di-methylation activity by MLL/SET complexes. PLoS Genet. 2013;9:e1003940. 24244196 10.1371/journal.pgen.1003940 PMC3828133

[45] Bode D, Yu L, Tate P, Pardo M, Choudhary J. Characterization of two distinct nucleosome remodeling and deacetylase (NuRD) complex assemblies in embryonic stem cells. Mol Cell Proteomics. 2016;15:878–91. 26714524 10.1074/mcp.M115.053207 PMC4813707

[46] Ee LS, McCannell KN, Tang Y, Fernandes N, Hardy WR, Green MR, et al. An embryonic stem cell-specific NuRD complex functions through interaction with WDR5. Stem Cell Reports. 2017;8:1488–96. 28528697 10.1016/j.stemcr.2017.04.020 PMC5470077

[47] Li Y, Han J, Zhang Y, Cao F, Liu Z, Li S, et al. Structural basis for activity regulation of MLL family methyltransferases. Nature. 2016;530:447–52. 26886794 10.1038/nature16952 PMC5125619

[48] Li Q, Huang Y, Xu J, Mao F, Zhou B, Sun L, et al. p53 inactivation unmasks histone methylation-independent WDR5 functions that drive self-renewal and differentiation of pluripotent stem cells. Stem Cell Reports. 2021;16:2642–58. 34715053 10.1016/j.stemcr.2021.10.002 PMC8581203

[49] Simonet T, Dulermo R, Schott S, Palladino F. Antagonistic functions of SET-2/SET1 and HPL/HP1 proteins in C. elegans development. Dev Biol. 2007;312:367–83. 17967446 10.1016/j.ydbio.2007.09.035

[50] Fisher K, Southall SM, Wilson JR, Poulin GB. Methylation and demethylation activities of a C. elegans MLL-like complex attenuate RAS signalling. Dev Biol. 2010;341:142–53. 20188723 10.1016/j.ydbio.2010.02.023

[51] Li T, Kelly WG. A role for Set1/MLL-related components in epigenetic regulation of the *Caenorhabditis elegans* germ line. PLoS Genet. 2011;7:e1001349. 21455483 10.1371/journal.pgen.1001349 PMC3063756

[52] Wang S, Fisher K, Poulin GB. Lineage specific trimethylation of H3 on lysine 4 during C. elegans early embryogenesis. Dev Biol. 2011;355:227–38. 21549110 10.1016/j.ydbio.2011.04.010

[53] Caron M, Gely L, Garvis S, Adrait A, Coute Y, Palladino F, et al. Loss of SET1/COMPASS methyltransferase activity reduces lifespan and fertility in *Caenorhabditis elegans*. Life Sci Alliance. 2022. 10.26508/lsa.202101140. 34893559 10.26508/lsa.202101140 PMC8675910

[54] Greer EL, Maures TJ, Ucar D, Hauswirth AG, Mancini E, Lim JP, et al. Transgenerational epigenetic inheritance of longevity in *Caenorhabditis elegans*. Nature. 2011;479:365–71. 22012258 10.1038/nature10572 PMC3368121

[55] Lee TW, David HS, Engstrom AK, Carpenter BS, Katz DJ. Repressive H3K9me2 protects lifespan against the transgenerational burden of COMPASS activity in *C. elegans*. Elife. 2019;8:e48498. 31815663 10.7554/eLife.48498 PMC7299346

[56] Zuryn S, Ahier A, Portoso M, White ER, Morin MC, Margueron R, et al. Transdifferentiation. Sequential histone-modifying activities determine the robustness of transdifferentiation. Science. 2014;345:826–9. 25124442 10.1126/science.1255885

[57] Abay-Norgaard S, Attianese B, Boreggio L, Salcini AE. Regulators of H3K4 methylation mutated in neurodevelopmental disorders control axon guidance in *C. elegans*. Development. 2020;147:dev190637. 32675280 10.1242/dev.190637 PMC7420840

[58] Kaser-Pebernard S, Muller F, Wicky C. LET-418/Mi2 and SPR-5/LSD1 cooperatively prevent somatic reprogramming of *C. elegans* germline stem cells. Stem Cell Rep. 2014;2:547–59. 10.1016/j.stemcr.2014.02.007 PMC3986580 24749077

[59] Robert VJ, Mercier MG, Bedet C, Janczarski S, Merlet J, Garvis S, et al. The SET-2/SET1 histone H3K4 methyltransferase maintains pluripotency in the *Caenorhabditis elegans* germline. Cell Rep. 2014;9:443–50. 25310986 10.1016/j.celrep.2014.09.018

[60] Ding W, Smulan LJ, Hou NS, Taubert S, Watts JL, Walker AK. S-Adenosylmethionine levels govern innate immunity through distinct methylation-dependent pathways. Cell Metab. 2015;22:633–45. 26321661 10.1016/j.cmet.2015.07.013 PMC4598287

[61] Greer EL, Maures TJ, Hauswirth AG, Green EM, Leeman DS, Maro GS, et al. Members of the H3K4 trimethylation complex regulate lifespan in a germline-dependent manner in *C. elegans*. Nature. 2010;466:383–7. 20555324 10.1038/nature09195 PMC3075006

[62] Brenner S. The genetics of *Caenorhabditis elegans*. Genetics. 1974;77:71–94. 4366476 10.1093/genetics/77.1.71 PMC1213120

[63] Liu T, Rechtsteiner A, Egelhofer TA, Vielle A, Latorre I, Cheung MS, et al. Broad chromosomal domains of histone modification patterns in *C. elegans*. Genome Res. 2011;21:227–36. 21177964 10.1101/gr.115519.110 PMC3032926

[64] Egan B, Yuan CC, Craske ML, Labhart P, Guler GD, Arnott D, et al. An alternative approach to ChIP-Seq normalization enables detection of genome-wide changes in histone H3 lysine 27 trimethylation upon EZH2 inhibition. PLoS ONE. 2016;11:e0166438. 27875550 10.1371/journal.pone.0166438 PMC5119738

[65] Ramirez F, Ryan DP, Gruning B, Bhardwaj V, Kilpert F, Richter AS, et al. deepTools2: a next generation web server for deep-sequencing data analysis. Nucleic Acids Res. 2016;44:W160-165. 27079975 10.1093/nar/gkw257 PMC4987876

[66] Gerstein MB, Lu ZJ, Van Nostrand EL, Cheng C, Arshinoff BI, Liu T, et al. Integrative analysis of the *C. elegans* genome by the modENCODE project. Science. 2010;330:1775–87. 21177976 10.1126/science.1196914 PMC3142569

[67] Chen RA, Down TA, Stempor P, Chen QB, Egelhofer TA, Hillier LW, et al. The landscape of RNA polymerase II transcription initiation in *C. elegans* reveals promoter and enhancer architectures. Genome Res. 2013;23:1339–47. 23550086 10.1101/gr.153668.112 PMC3730107

[68] Janes J, Dong Y, Schoof M, Serizay J, Appert A, Cerrato C, et al. Chromatin accessibility dynamics across *C. elegans* development and ageing. Elife. 2018;7:e37344. 30362940 10.7554/eLife.37344 PMC6231769

[69] Bolger AM, Lohse M, Usadel B. Trimmomatic: a flexible trimmer for Illumina sequence data. Bioinformatics. 2014;30:2114–20. 24695404 10.1093/bioinformatics/btu170 PMC4103590

[70] Kim D, Pertea G, Trapnell C, Pimentel H, Kelley R, Salzberg SL. TopHat2: accurate alignment of transcriptomes in the presence of insertions, deletions and gene fusions. Genome Biol. 2013;14:R36. 23618408 10.1186/gb-2013-14-4-r36 PMC4053844

[71] Anders S, Pyl PT, Huber W. HTSeq--a Python framework to work with high-throughput sequencing data. Bioinformatics. 2015;31:166–9. 25260700 10.1093/bioinformatics/btu638 PMC4287950

[72] Love MI, Huber W, Anders S. Moderated estimation of fold change and dispersion for RNA-seq data with DESeq2. Genome Biol. 2014;15:550. 25516281 10.1186/s13059-014-0550-8 PMC4302049

[73] Livak KJ, Schmittgen TD. Analysis of relative gene expression data using real-time quantitative PCR and the 2(-Delta Delta C(T)) method. Methods. 2001;25:402–8. 11846609 10.1006/meth.2001.1262

[74] Orlando DA, Chen MW, Brown VE, Solanki S, Choi YJ, Olson ER, et al. Quantitative ChIP-Seq normalization reveals global modulation of the epigenome. Cell Rep. 2014;9:1163–70. 25437568 10.1016/j.celrep.2014.10.018

[75] Attianese B, Wang H, Madsen K, Zeijdner M, Helin K, Abay-Norgaard S, et al. Functional dissection of H3K4 methyltransferases reveals distinct catalytic and non-catalytic roles in *C. elegans* development. Development. 2025;152:dev204924. 41122790 10.1242/dev.204924 PMC12633793

[76] Vandamme J, Lettier G, Sidoli S, Di Schiavi E, Norregaard Jensen O, Salcini AE. The *C. elegans* H3K27 demethylase UTX-1 is essential for normal development, independent of its enzymatic activity. PLoS Genet. 2012;8:e1002647. 22570628 10.1371/journal.pgen.1002647 PMC3342935

[77] Holdorf AD, Higgins DP, Hart AC, Boag PR, Pazour GJ, Walhout AJM, et al. WormCat: an online tool for annotation and visualization of *Caenorhabditis elegans* genome-scale data. Genetics. 2020;214:279–94. 31810987 10.1534/genetics.119.302919 PMC7017019

[78] Ayoub A, Park SH, Lee YT, Cho US, Dou Y. Regulation of MLL1 methyltransferase activity in two distinct nucleosome binding modes. Biochemistry. 2022;61:1–9. 34928138 10.1021/acs.biochem.1c00603

[79] Park SH, Ayoub A, Lee YT, Xu J, Kim H, Zheng W, et al. Cryo-EM structure of the human MLL1 core complex bound to the nucleosome. Nat Commun. 2019;10:5540. 31804488 10.1038/s41467-019-13550-2 PMC6895043

[80] Mittal A, Hobor F, Zhang Y, Martin SR, Gamblin SJ, Ramos A, et al. The structure of the RbBP5 beta-propeller domain reveals a surface with potential nucleic acid binding sites. Nucleic Acids Res. 2018;46:3802–12. 29897600 10.1093/nar/gky199 PMC6283417

[81] Xiao Y, Bedet C, Robert VJ, Simonet T, Dunkelbarger S, Rakotomalala C, et al. *Caenorhabditis elegans* chromatin-associated proteins SET-2 and ASH-2 are differentially required for histone H3 Lys 4 methylation in embryos and adult germ cells. Proc Natl Acad Sci U S A. 2011;108:8305–10. 21527717 10.1073/pnas.1019290108 PMC3100940

[82] Wysocka J, Swigut T, Milne TA, Dou Y, Zhang X, Burlingame AL, et al. WDR5 associates with histone H3 methylated at K4 and is essential for H3 K4 methylation and vertebrate development. Cell. 2005;121:859–72. 15960974 10.1016/j.cell.2005.03.036

[83] Fischer V, Plassard D, Ye T, Reina-San-Martin B, Stierle M, Tora L, et al. The related coactivator complexes SAGA and ATAC control embryonic stem cell self-renewal through acetyltransferase-independent mechanisms. Cell Rep. 2021;36:109598. 34433046 10.1016/j.celrep.2021.109598 PMC8430043

[84] Shi X, Hong T, Walter KL, Ewalt M, Michishita E, Hung T, et al. ING2 PHD domain links histone H3 lysine 4 methylation to active gene repression. Nature. 2006;442:96–9. 16728974 10.1038/nature04835 PMC3089773

[85] Xie Z, Chai Y, Zhu Z, Shen Z, Guo Z, Zhao Z, et al. Vacuolar H(+)-ATPase determines daughter cell fates through asymmetric segregation of the nucleosome remodeling and deacetylase complex. Elife. 2024;12:RP89032. 38994733 10.7554/eLife.89032 PMC11245309

[86] Kulkarni SS, Khokha MK. WDR5 regulates left-right patterning via chromatin-dependent and -independent functions. Development. 2018;145:dev159889. 30377171 10.1242/dev.159889 PMC6288385

[87] Li T, Kelly WG. A role for WDR5 in TRA-1/Gli mediated transcriptional control of the sperm/oocyte switch in *C. elegans*. Nucl Acid Res. 2014;42:5567–81. 10.1093/nar/gku221 PMC4027197 24682813

[88] Huang Y, Jay KL, Huang AY, Wan J, Jangam SV, Chorin O, et al. Loss-of-function in RBBP5 results in a syndromic neurodevelopmental disorder associated with microcephaly. Genet Med. 2024;26:101218. 39036895 10.1016/j.gim.2024.101218 PMC11648989

[89] Kamath RS, Fraser AG, Dong Y, Poulin G, Durbin R, Gotta M, et al. Systematic functional analysis of the *Caenorhabditis elegans* genome using RNAi. Nature. 2003;421:231–7. 12529635 10.1038/nature01278

[90] Mazzetto M, Gonzalez LE, Sanchez N, Reinke V. Characterization of the distribution and dynamics of chromatin states in the *C. elegans* germline reveals substantial H3K4me3 remodeling during oogenesis. Genome Res. 2024;34:57–69. 38164610 10.1101/gr.278247.123 PMC10903938

[91] Greer EL, Beese-Sims SE, Brookes E, Spadafora R, Zhu Y, Rothbart SB, et al. A histone methylation network regulates transgenerational epigenetic memory in *C. elegans*. Cell Rep. 2014;7:113–26. 24685137 10.1016/j.celrep.2014.02.044 PMC4012616

[92] Collins BE, Greer CB, Coleman BC, Sweatt JD. Histone H3 lysine K4 methylation and its role in learning and memory. Epigenetics Chromatin. 2019;12:7. 30616667 10.1186/s13072-018-0251-8 PMC6322263

[93] Howe FS, Fischl H, Murray SC, Mellor J. Is H3K4me3 instructive for transcription activation? BioEssays. 2017;39:1–12. 28004446 10.1002/bies.201600095

[94] Hodl M, Basler K. Transcription in the absence of histone H3.2 and H3K4 methylation. Curr Biol. 2012;22:2253–7. 23142044 10.1016/j.cub.2012.10.008

[95] Zylicz JJ, Bousard A, Zumer K, Dossin F, Mohammad E, da Rocha ST, et al. The implication of early chromatin changes in X chromosome inactivation. Cell. 2019;176(182–197):e123. 10.1016/j.cell.2018.11.041 PMC6333919 30595450

[96] Pferdehirt RR, Kruesi WS, Meyer BJ. An MLL/COMPASS subunit functions in the *C. elegans* dosage compensation complex to target X chromosomes for transcriptional regulation of gene expression. Genes Dev. 2011;25:499–515. 21363964 10.1101/gad.2016011 PMC3049290

