# Supplementary figures and images for: WDR-5 exhibits H3K4 methylation-independent activity during embryonic development in C. elegans
Source: Epigenetics Chromatin. 2026 Mar 25;19:19. doi: 10.1186/s13072-026-00669-y (PMC13141272; doi:10.1186/s13072-026-00669-y)

Suppl. Figure 1

Wild Type N2

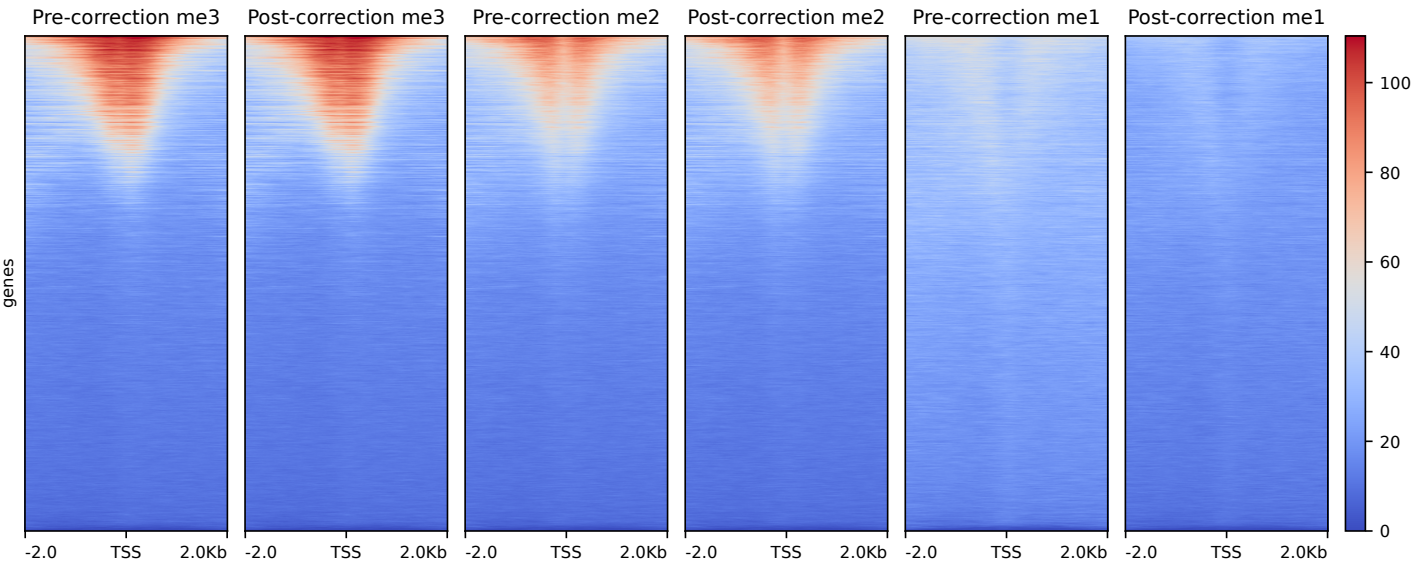

*wdr-5(-)*

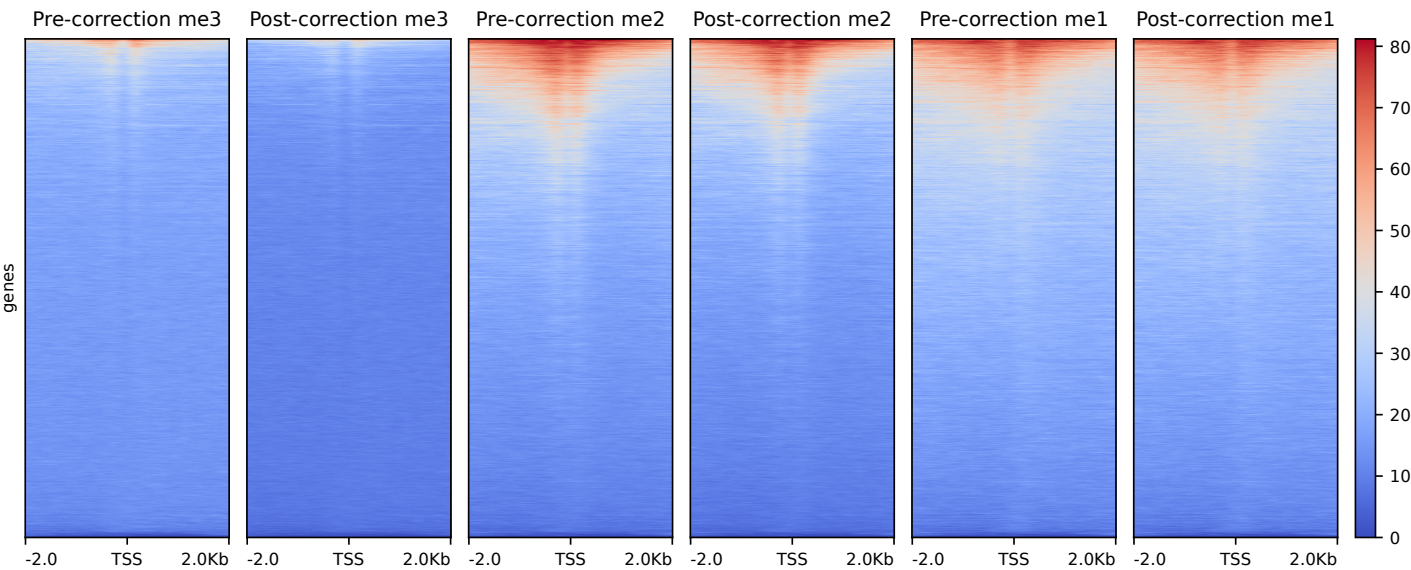

*rbbp-5 (-)*

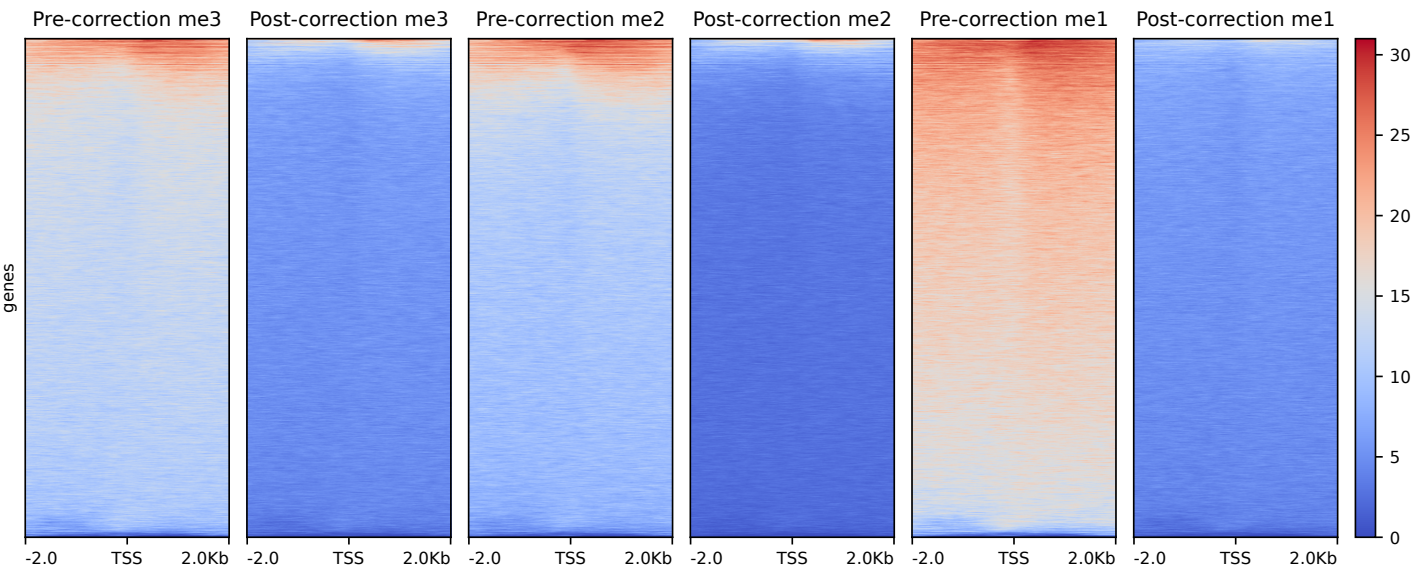

Supplement: Supplementary file 1 — Supplementary Material 1: Figure 1. Effects of normalisation on H3K4 methylation profiles. A Heatmaps showing histone H3K4 methylation ChIP-seq signal intensities centred on TSS (±2 kb) in wild-type (N2), wdr-5(-), and rbbp-5(-) mutants. For each genotype pre-correction (raw) and post-correction (normalised) ChIP-seq signals are shown for H3K4me3, me2, and me1. [file 13072_2026_669_MOESM1_ESM.pdf]

Suppl. Figure 2

A

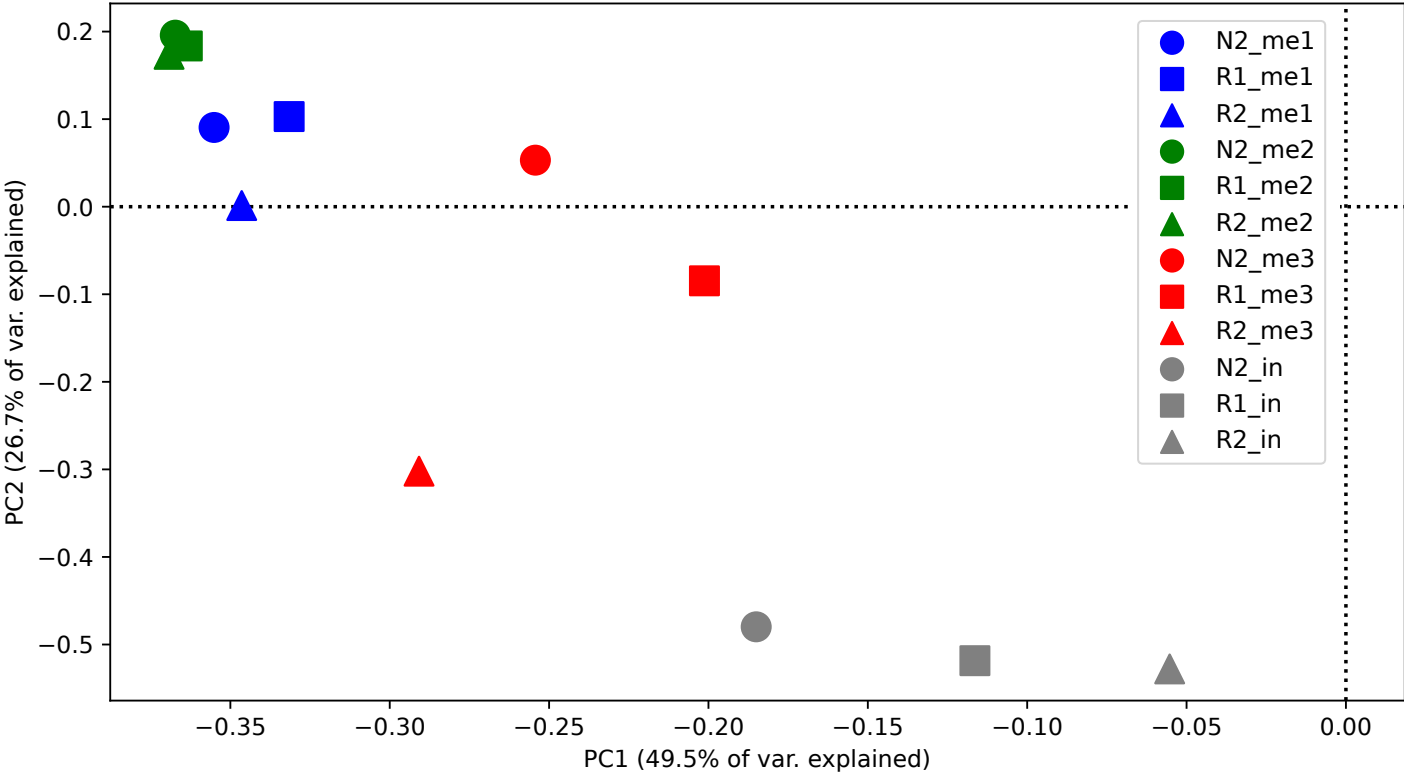

B

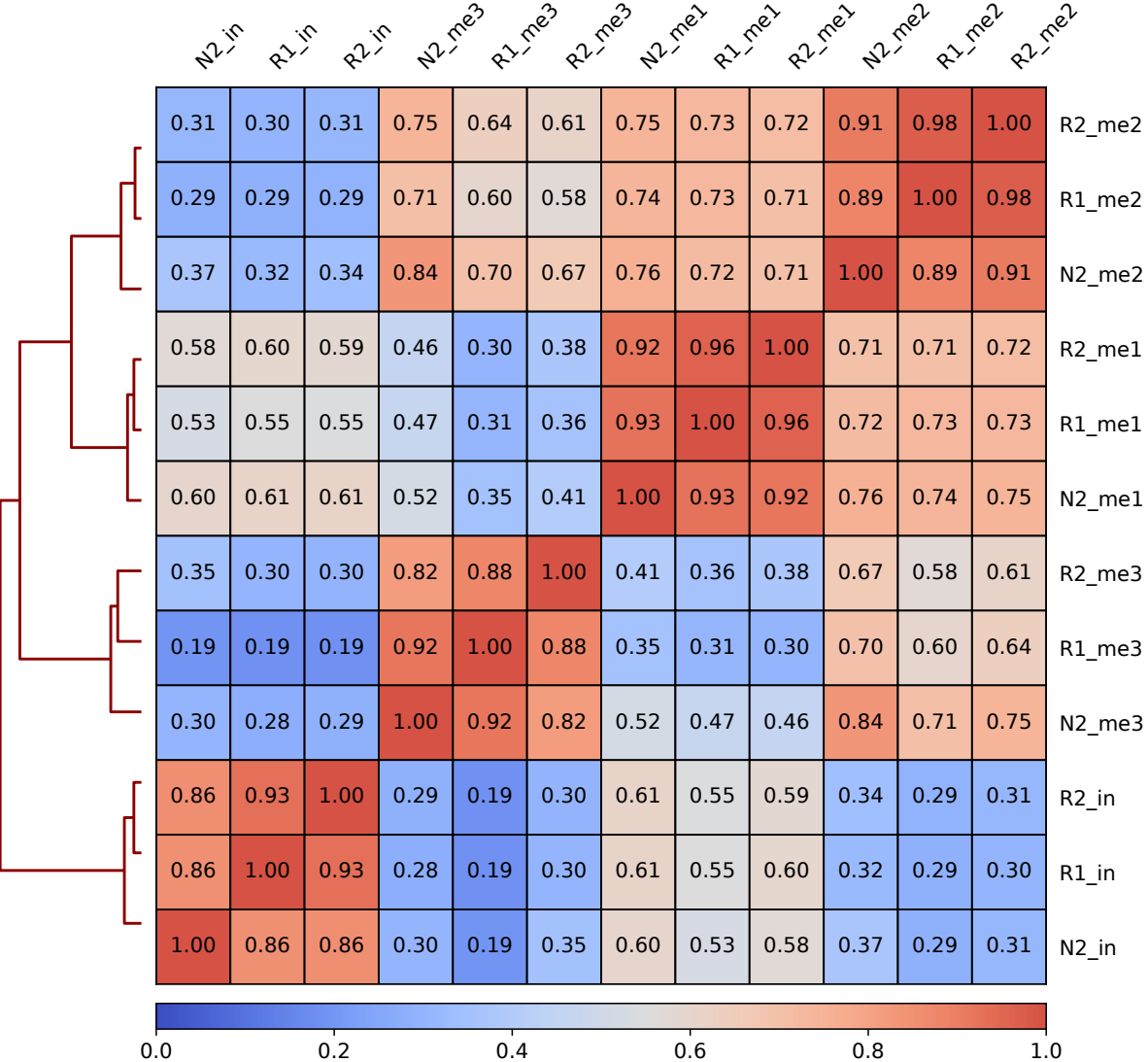

Supplement: Supplementary file 2 — Supplementary Material 2: Figure 2. Benchmarking spike-in ChIP-seq datasets against modENCODE using PCA and Pearson correlation analysis. A Principal Component Analysis (PCA) of usable ChIP-seq tags from this study (N2) and public C. elegans datasets from modENCODE (R1, R2) for H3K4me1, H3K4me2, and H3K4me3. Samples cluster primarily by histone modification (color-coded) and by replicate, indicating high signal specificity and low technical noise. B Pearson correlation matrix of usable ChIP-seq tags shows high intra-group correlation, especially among replicates targeting the same modification. Hierarchical clustering groups samples by H3K4me1, H3K4me2, and H3K4me3, with datasets from this study (N2) and modENCODE (R1, R2) generally in strong agreement. [file 13072_2026_669_MOESM2_ESM.pdf]

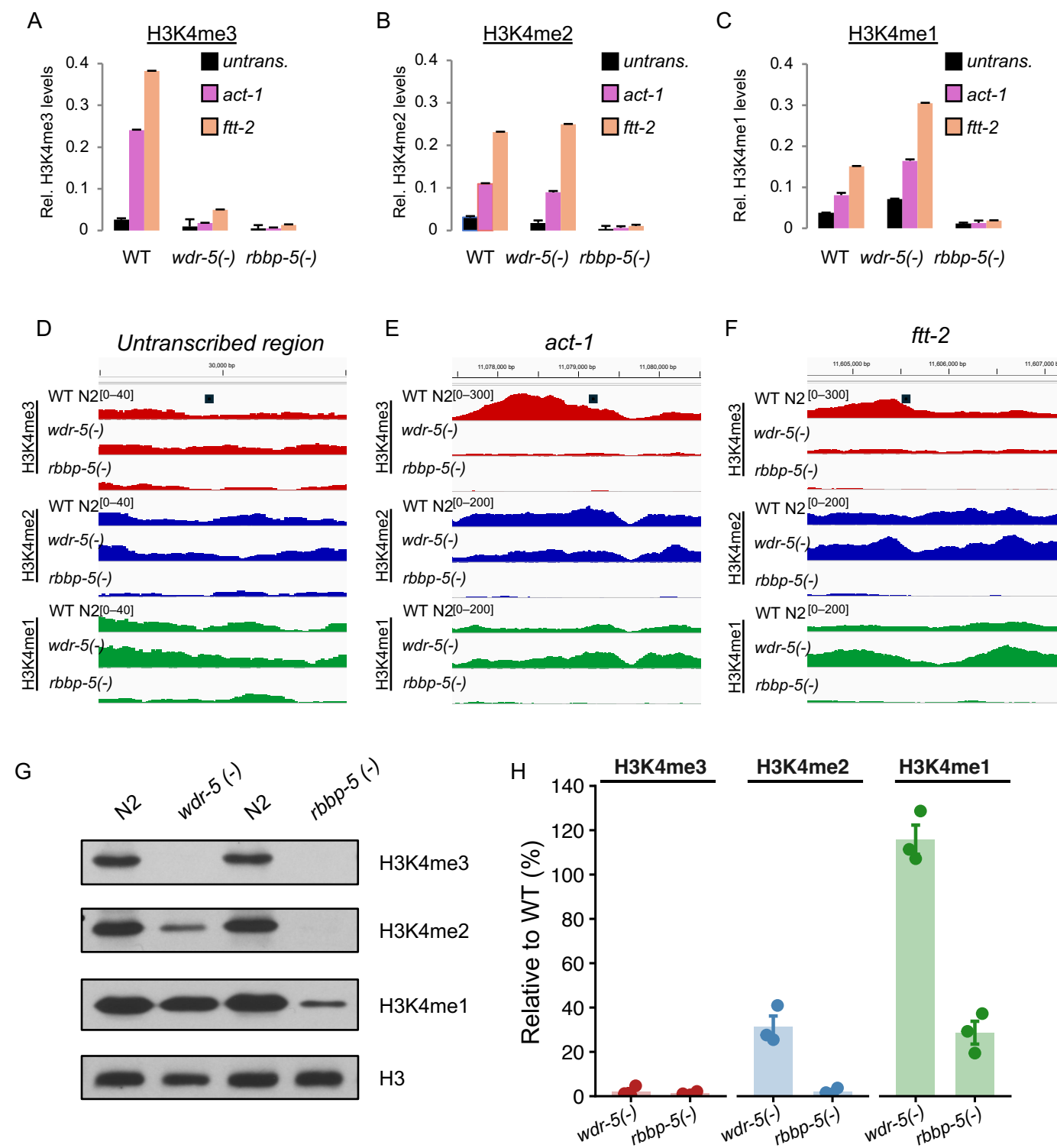

Supplement: Supplementary file 3 — Supplementary Material 3: Figure 3. ChIP-qPCR with associated track and Western blots showing broad agreement with the spike-in-normalised ChIP-seq data. (A-C) ChIP-qPCR at three loci (an untranscribed region, act-1 and ftt-2) for H3K4me3/me2/me1 into wild type (WT) embryos, wdr-5(-) and rbbp-5(-) mutants. (D-F) Associated track from the spike-in-normalised ChIP-seq data. H3K4me3/me2/me1 are colour coded in red, blue and green respectively and genotype indicated on the left. The black box indicates approximate location of the primer pairs (see Table S8 for exact location) and the top-left values within square brackets are signal levels selection. (G-H) Western blots on mixed stage embryos from wild type (N2), wdr-5(-) and rbbp-5(-) mutants for H3K4me3/me2/me1 normalised against H3 and performed three times independently. Error bars are S.E.M. [file 13072_2026_669_MOESM3_ESM.pdf]

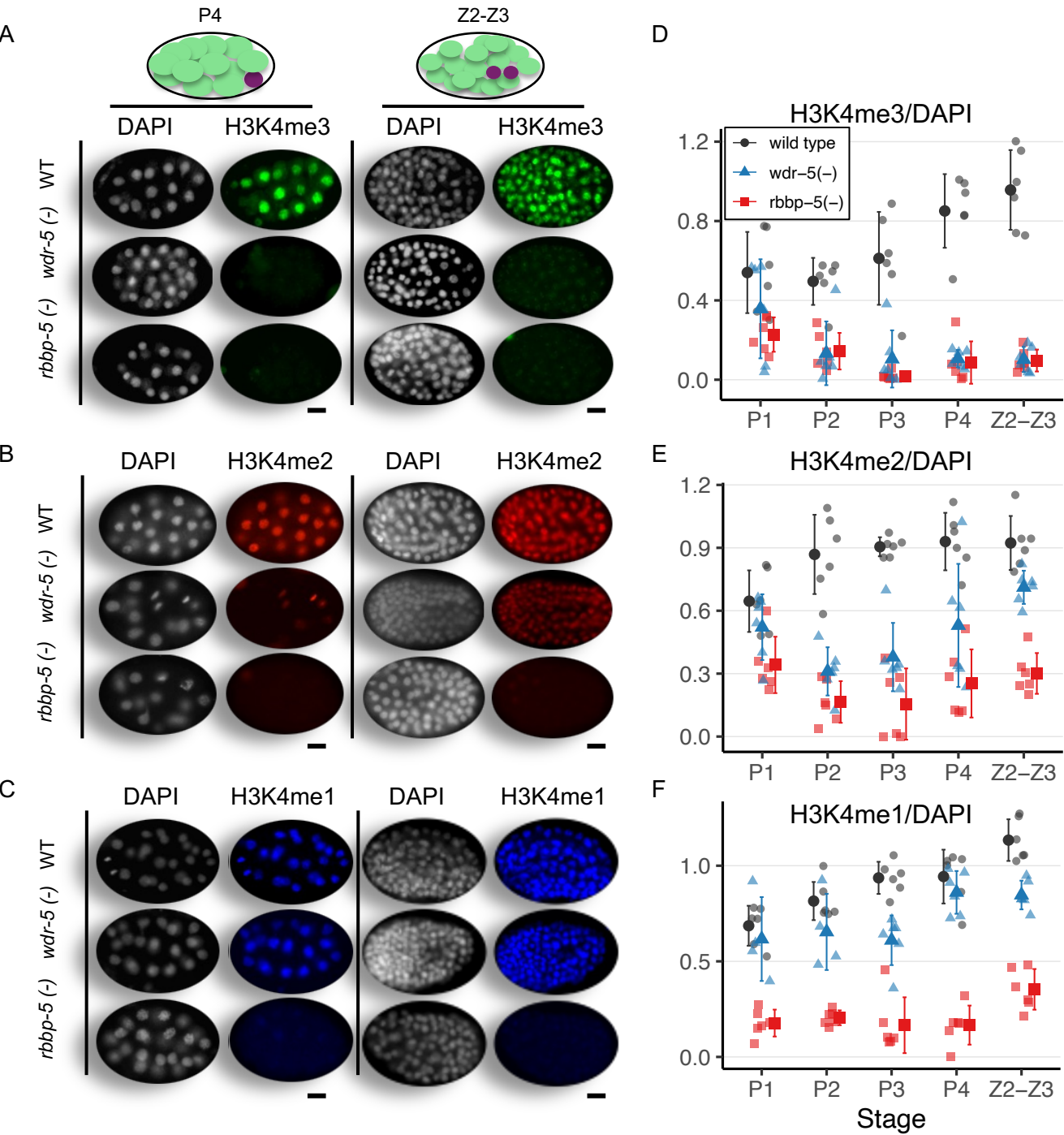

Supplement: Supplementary file 4 — Supplementary Material 4: Figure 4. Immuno-fluorescence staining against H3K4me3/me2/me1 and DAPI staining for the indicated genotype has been performed on embryos from the P1 stage (two-cell) to Z2-Z3 showing that loss of WDR-5 is strikingly preventing H3K4me2 deposition at early stages of embryogenesis, but only partially at late stages of embryogenesis. (A-C) Photographs of representative immuno-fluorescence staining at P4 and Z2-Z3. Scale bar is 10μm and indicated to the bottom right. (D-F) The abundance of each mark in the indicated genotype and developmental stages have been calculated from quantifying 6 to 8 cells for each stage and normalising the H3K4 methylation signal by DAPI signal intensity. [file 13072_2026_669_MOESM4_ESM.pdf]

Suppl. Figure 5

A

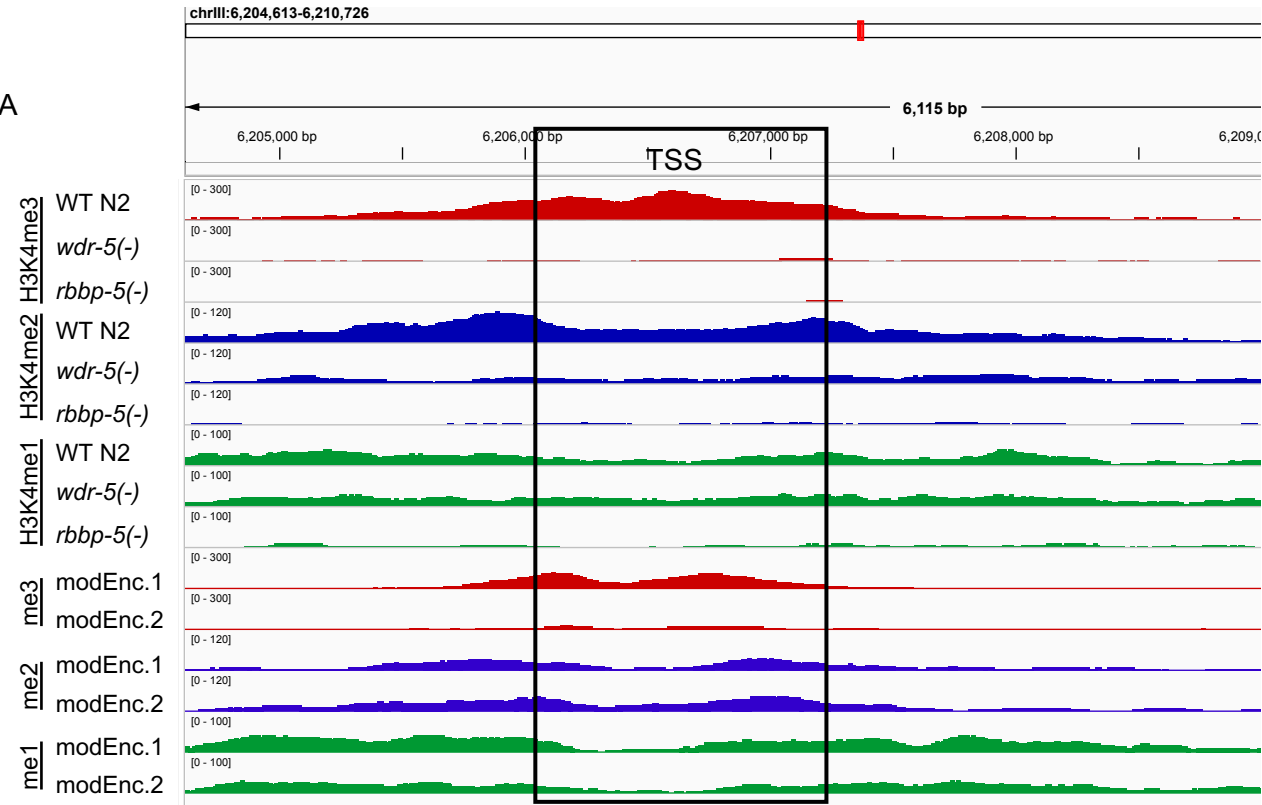

B

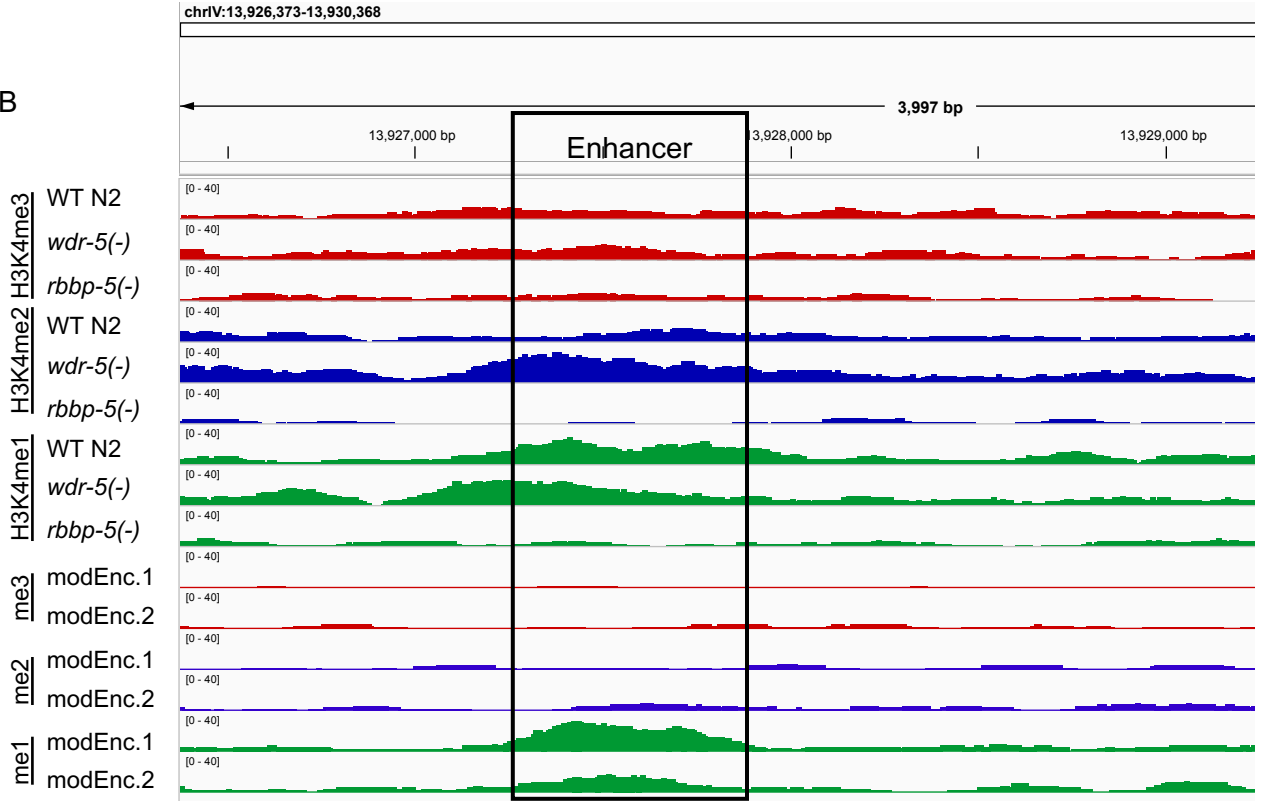

Supplement: Supplementary file 5 — Supplementary Material 5: Figure 5. IGV tracks from spike-in-normalised ChIP-seq as well as modEncode data. (A-B) Comparisons between this study ChIP-seq data and modENCODE data as well as showing the effects that loss of WDR-5 and RBBP-5 have on H3K4me3/me2/me1 at a TSS and an enhancer at loci indicated at the top left. The box indicate the approximate location of the TSS or enhancer as defined in our study. [file 13072_2026_669_MOESM5_ESM.pdf]

Suppl. Figure 6

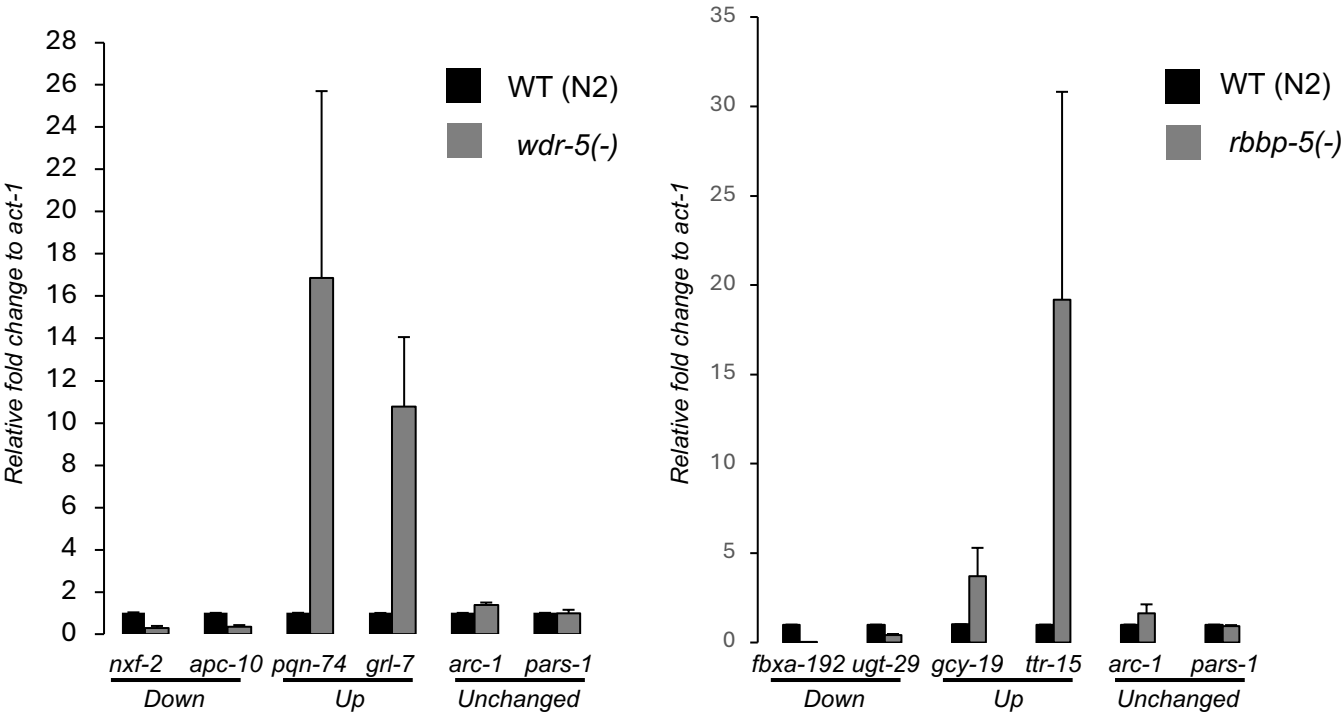

Supplement: Supplementary file 6 — Supplementary Material 6: Figure 6. Quantitative RT-PCR in wdr-5(-) and rbbp-5(-) mutants on selected downregulated, upregulated genes as well as on genes displaying unchanged levels of expression correlate with RNA-seq data. Experiments were performed in triplicates on separate biological samples and normalised using act-1 and the ΔΔct method. The error bars represent the ±SEM. [file 13072_2026_669_MOESM6_ESM.pdf]

Suppl. Figure 7

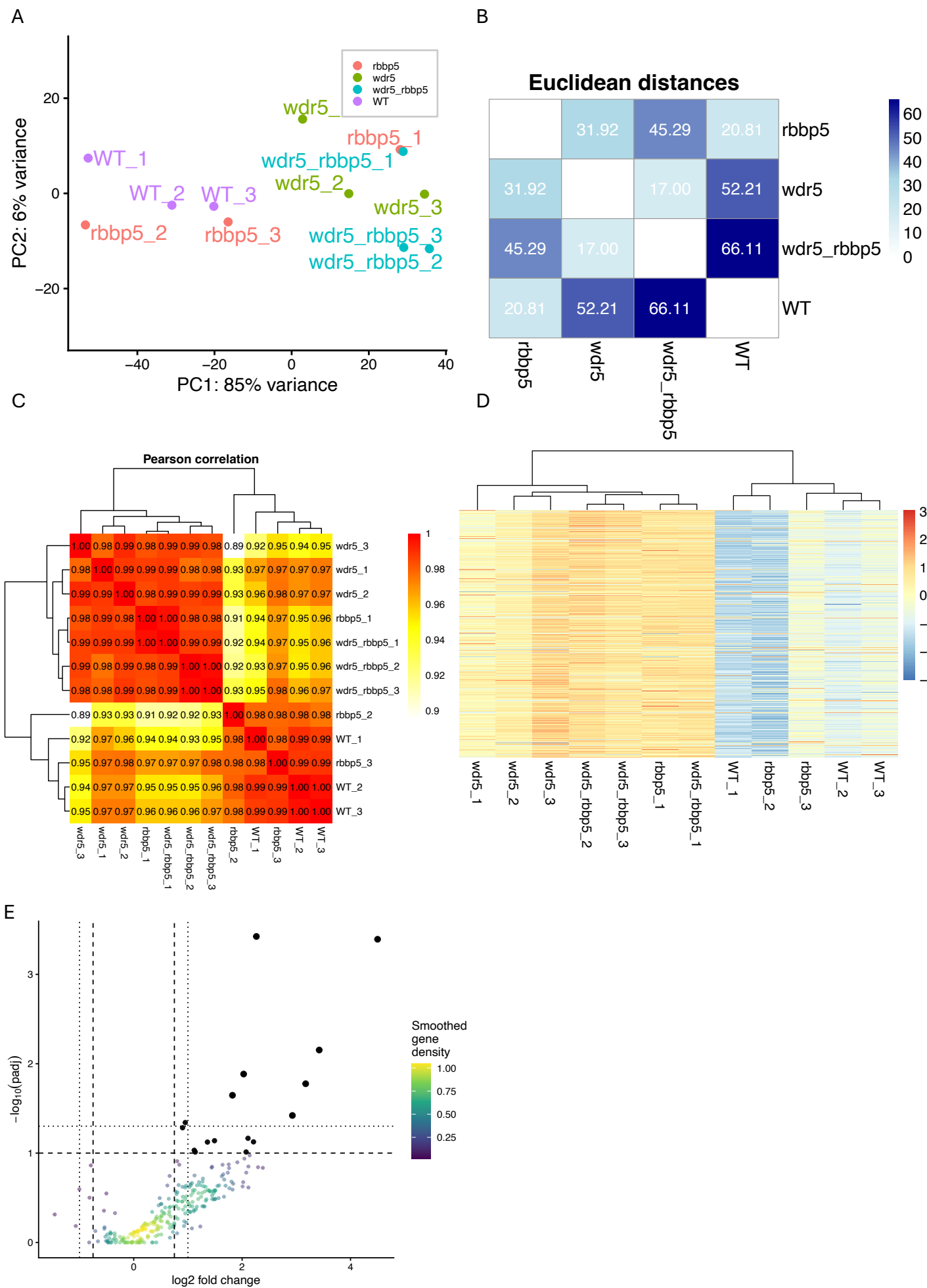

Supplement: Supplementary file 7 — Supplementary Material 7: Figure 7. Analysis of RNA-seq data showing that the transcriptome of the single wdr-5(-) mutant is closer to the double rbbp-5(-);wdr-5(-) mutant. (A) PCA shows that most samples cluster according to their respective genotype except for replicate rbbp-5(-)_1. (B) Euclidean distances show that the single wdr-5(-) mutant is closer to the double rbbp-5(-);wdr-5(-) mutant when compared with the distance between the single rbbp-5(-) mutant and the double rbbp-5(-);wdr-5(-) mutant, (17 versus 45.29). (C) Pearson correlation shows that wdr-5(-) and the double rbbp-5(-);wdr-5(-) form a distinct cluster whereas wild type and two replicates of rbbp-5(-) another. (D) Hierarchical clustering shows that wdr-5(-) and the double rbbp-5(-);wdr-5(-) form a distinct cluster whereas wild type and two replicates of rbbp-5(-) another. (E) Cook’s analysis demonstrating the limited influence of the rbbp-5_1 replicate on differential expression results. After expression filtering, 277 Cook’s-sensitive genes (Cook’s >1) were retained. Of these, only 7 met our standard differential expression criteria, and a total of 16 met relatively more relaxed criteria. Points are coloured according to smoothed gene density (two-dimensional kernel density estimate). Dashed and dotted lines indicate relaxed (|log2FC > 0.75 and p-adj < 0.1) as well as thresholds used throughout our study (|log2FC >1| and p-adj < 0.05), respectively. [file 13072_2026_669_MOESM7_ESM.pdf]

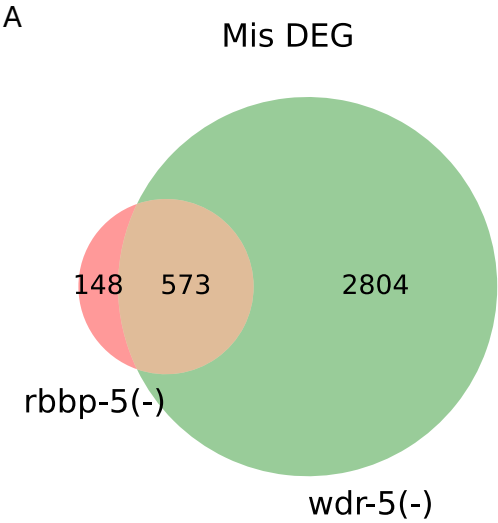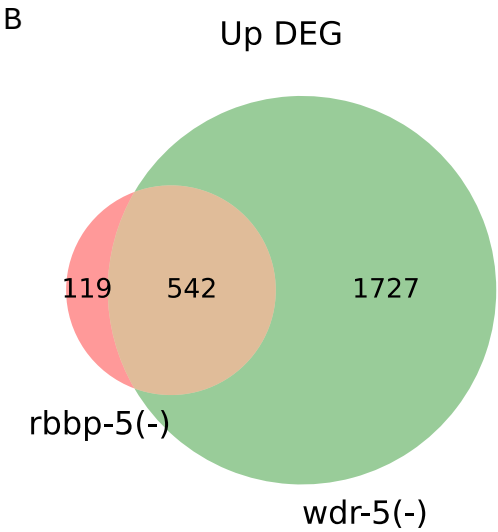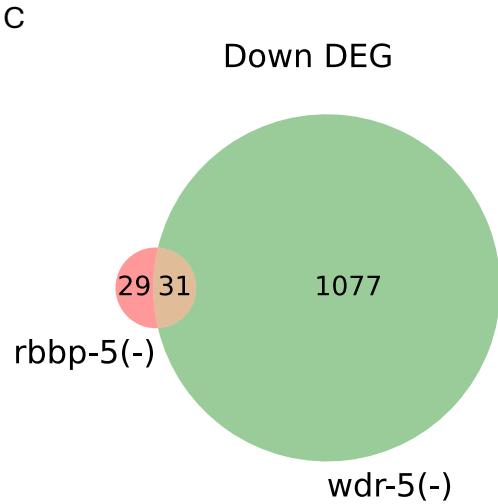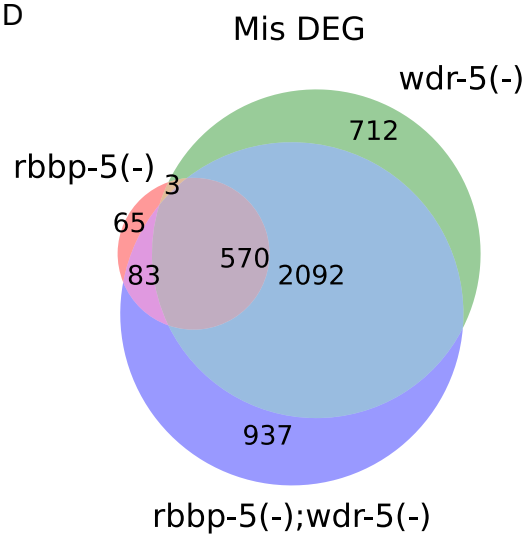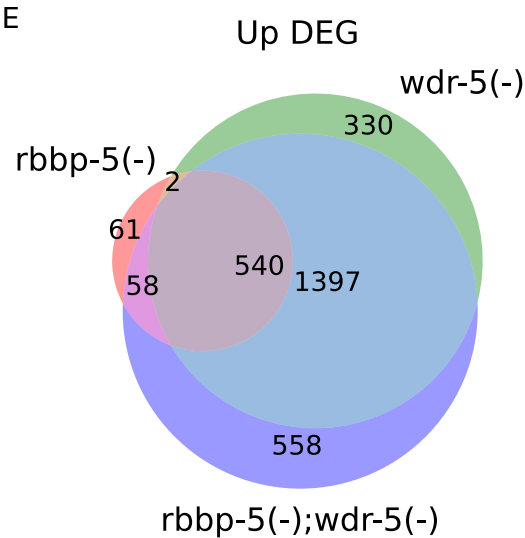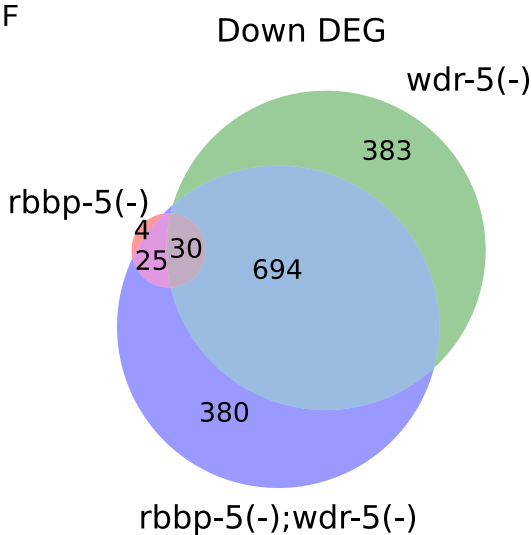

Supplement: Supplementary file 8 — Supplementary Material 8: Figure 8. Venn diagrams (2-way and 3-way) showing that WDR-5 and RBBP-5 are part of the SET/MLL complex. (A-C) 2-way Venn diagrams showing the overlap of mis-, up- and downregulated genes between the rbbp-5(-) and wdr-5(-) single mutants. (D-F) 3-way Venn diagrams showing the overlap of mis-, up- and downregulated between rbbp-5(-), wdr-5(-), and rbbp-5(-); wdr-5(-) mutants. [file 13072_2026_669_MOESM8_ESM.pdf]

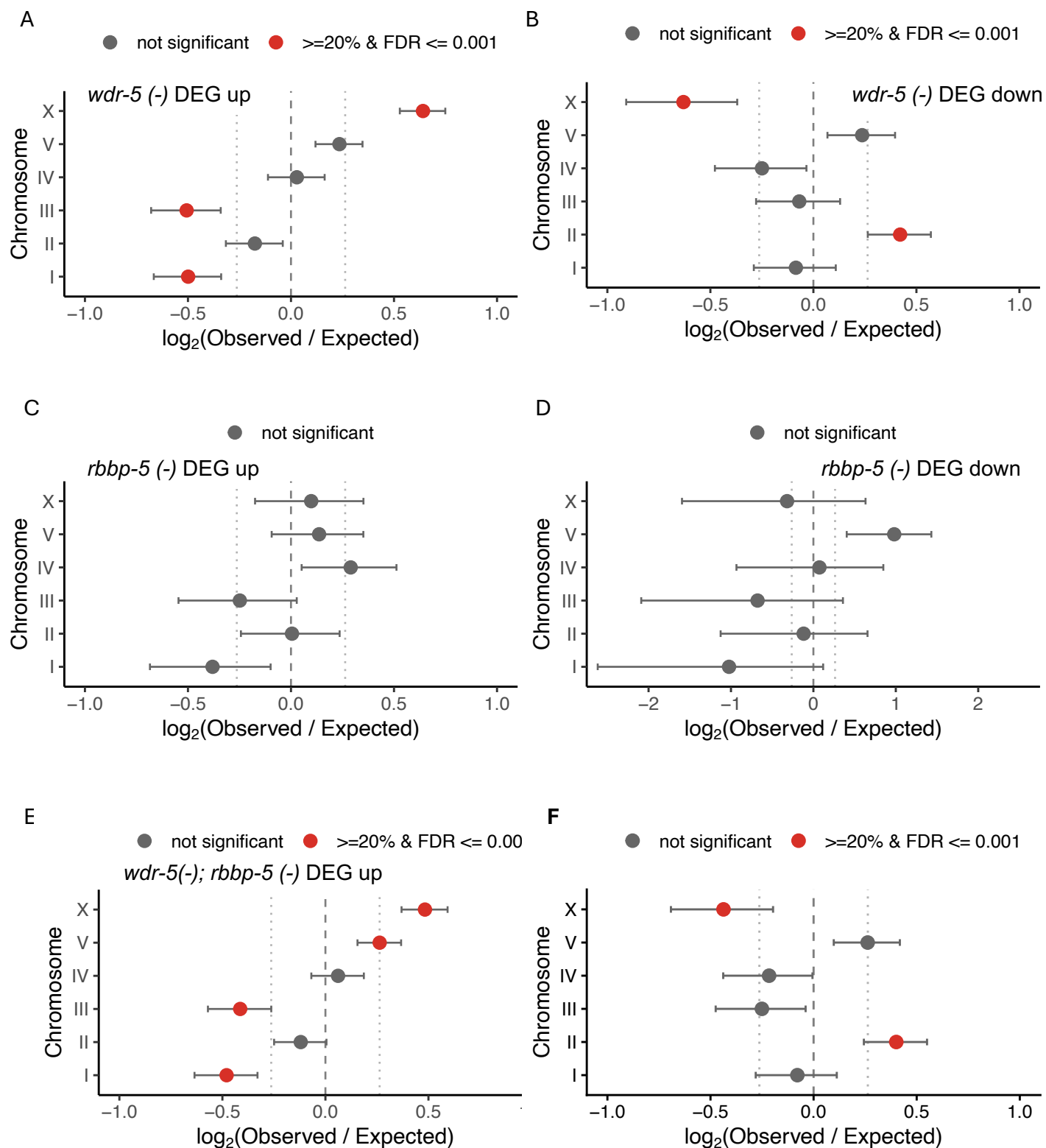

Supplement: Supplementary file 9 — Supplementary Material 9: Figure 9. Enrichment plots for chromosomal analysis of RNA-deq data showing that DEG on chromosome X are over-represented for upregulated genes but under-represented for downregulated genes in the single wdr-5(-) and double rbbp-5(-);wdr-5(-) mutants, but not in rbbp-5(-). For each mutant and gene-expression direction (up or down), log2 (observed/expected) enrichment values were plotted on the x-axis, with chromosomes (I–V, X) on the y-axis. Error bars represent 95 % confidence intervals of the observed proportion. Vertical dashed lines indicate the random expectation (0) and dotted lines mark the ±20 % biological threshold (±log2 1.2 ≈ ±0.26). Points were coloured red when both criteria (FDR ≤ 0.01 and ≥ 20 % enrichment) were satisfied, and grey otherwise. (A-B) Forest plots style for up- or downregulated genes (wdr-5(-) DEG up or wdr-5(-) DEG down ) for each chromosome (C-D) Forest plots style for up- or downregulated genes (rbbp-5(-) DEG up or rbbp-5(-) DEG down ) for each chromosome. (E-F) Forest plots style for up- or downregulated genes (rbbp-5(-);wdr-5(-) DEG up or rbbp-5(-);wdr-5(-) DEG down ) for each chromosome. [file 13072_2026_669_MOESM9_ESM.pdf]
